# Experiences and perceptions of men following breast cancer diagnosis: a mixed method systematic review

**DOI:** 10.1186/s12885-024-11911-9

**Published:** 2024-02-06

**Authors:** Mary Abboah-Offei, Jonathan Bayuo, Yakubu Salifu, Oladayo Afolabi, Theophilus N. Akudjedu

**Affiliations:** 1 School of Health and Social Care, Edinburgh Napier University, Sighthill Court, Sighthill Campus, Edinburgh, UK; 2 School of Nursing, The Hong Kong Polytechnic University, Hong Kong Special Administrative Region, Hongkong, China; 3 International Observatory On End of Life Care (IOELC), Faculty of Health and Medicine, Division of Health Research, Lancaster University, Lancaster, LA1 4AT UK; 4 Florence Nightingale Faculty of Nursing, Midwifery and Palliative Care, King’s College London, London, WC2R 2LS UK; 5 Institute of Medical Imaging & Visualisation, Department of Medical Science & Public Health, Faculty of Health & Social Science, Bournemouth University, Bournemouth, UK

**Keywords:** Male breast cancer, Experiences, Perceptions, Treatment approaches, Systematic review, Masculinity

## Abstract

**Background:**

Men with breast cancer experience unique physical and emotional challenges. However, a thorough understanding of these experiences including the psychosocial effects and supportive care needs have received less attention. In some settings, men with breast cancer experience stigma within the healthcare system and their care needs are not prioritised. This influences the level of professional support offered, consequently worsening their health and well-being outcomes. This review explored the variabilities in the experiences and treatment modalities of male breast cancer (MBC) across different contexts.

**Methods:**

All primary study designs including qualitative, quantitative, and mixed methods studies that reported on the experiences, treatment approaches and outcomes of MBC were included in this systematic review. Six databases (Embase, Medline, PsycINFO, Global Health, CINAHL and Web of Science) were searched for articles from January 2000 to September 2023. A results-based convergence synthesis was used for data analysis and reported using PRISMA guidelines.

**Results:**

Of the studies screened (*n* = 29,687), forty-four fulfilled the predetermined criteria and were included. Our findings relating to the experiences and treatment approaches of MBC are broadly themed into three parts. *Theme 1—Navigating through a threat to masculinity:* describes how males experienced the illness reflecting on detection, diagnosis, coming to terms with breast cancer, and disclosure. *Theme 2- Navigating through treatment:* captures the experiences of undergoing breast cancer treatment/ management following their diagnosis. *Theme 3—Coping and support systems:* describes how MBC patients coped with the disease, treatment process, aftercare/rehabilitative care, and the available support structures.

**Conclusions:**

Men experience a myriad of issues following a breast cancer diagnosis, especially with their masculinity. Awareness creation efforts of MBC among the public and healthcare practitioners are urgently required, which could change the perception of men in promoting early diagnosis, adherence to treatments, post-treatment monitoring, oncological results and a better quality of life. Considerations for training, education and development of specialised guidelines for healthcare practitioners on MBC would provide the necessary knowledge and skills to enhance their practice through the adoption of person-centred and male-specific care strategies. Professional care intervention and support for MBC should not end after the diagnosis phase but should extend to the entire treatment continuum and aftercare including future research focusing on MBC specific clinical trials.

**Trial registration:**

PROSPERO Registration No. CRD42021228778.

## Background

Male breast cancer (MBC) is a rare condition, accounting for less than 1% of all breast cancers. About 2,710 men are estimated to be diagnosed with breast cancer, with approximately 530 men projected to die from breast cancer in 2022 and have about 1 in 833 lifetime risk of being diagnosed with the disease in the United States [1]. Data from the Global Burden of Disease 2017 database indicate that the incidence of MBC increased from 8.5 thousand in 1990 to 23.1 thousand in 2017 with 123 countries showing a significant increasing trend in MBC incidence rates [2]. There are variations in the incidence of MBC among countries for instance, in Thailand MBC incidence was lower than that in Israel, and the rate of variability has been attributed to population-specific factors [3]. Additionally, disparities have been noted in the incidence, prevalence, mortality, and burden of cancer and related adverse health conditions in specific population groups [4]. Some of these disparities have been noted in the United States, where black men are reported to have higher incidence and mortality rates compared to white men in the context of all cancer [4–6].

Evidence suggests that MBC is mostly diagnosed late (49%) when the disease is more advanced compared to women (33%) leading to relatively worse prognosis [7–11]. This has been attributed to delayed presentation, lack of screening, reduced awareness by treating providers and a lack of awareness of the disease among men [12–15]. Consequently, MBCs are mainly diagnosed with more severe clinical manifestations with relatively complex tumour characteristics (i.e., larger sizes and extensive lymph node involvement) [16], associated with higher proportions of positive hormone receptors, which mostly results in prolonged treatment delay, and metastasis of the disease at diagnosis compared to female breast cancer [17]. This has been influenced by issues with lower socioeconomic status, barriers to accessing healthcare and insurance cover issues in the context of the United States, adherence to treatment, post-treatment follow-up, and stigma [7, 18–20]. MBC patients suffer from a triple stigma including stigma by healthcare professionals, society, and especially by themselves as they struggle to accept the disease which has been labelled as a woman's disease [20].

Treatment for MBC has mainly been informed by available evidence for female breast cancer [21], and no randomised data exists for optimal management strategies for men including surgery, systemic therapy, and radiation [22]. Some guidelines have been published for the management of MBC [23–25]; however, these guidelines are rarely based on clinical trials leading to a paucity of literature on the evaluation of outcomes for MBC. According to Corrigan et al. [26], of the 131 breast cancer clinical trials conducted, there was only 0.087% of male patients represented among study participants.

Moreover, MBC being widely described as a 'woman’s disease' has psychosocially impacted the experience of men in terms of their body image and appearance as well as masculinity [27, 28]. A critical psychosocial problem for MBC patients is concerns with body image [29], because both the disease and its treatment can lead to significant alterations to their looks and how the body functions [30]. With masculinity often associated with chest rather than breast [31–33], being linked to a “woman’s disease” attributed to the body part that men do not relate to is probably threatening their masculinity [34]. Men with breast cancer also face unique physical and emotional challenges however, there is inconclusive understanding of men’s experiences of the psychosocial implications of MBC as well as the supportive care needs [35, 36]. Therefore, in this review, we explored the experiences of MBC patients and the management approaches across different demographic contexts.

## Methods

### Review question

What are the experiences and perceptions of MBC patients following diagnosis?

### Design

We conducted a mixed method systematic review with an interpretive and inductive stance [37] and reported in line with the Preferred Reporting Items for Systematic Review and Meta-Analysis (PRISMA) guidelines [38].

### Search strategy

We identified relevant studies through a search in six electronic databases: Global Health, CINAHL, Medline, PsycINFO, Embase, and Web of Science. Furthermore, we searched reference lists of included studies for additional studies. The search duration in these databases covered January 2000 to December 2023, and was updated in September 2023.

A combination of the following keywords was used for search strategy i) ‘Men’ OR ‘Male’ OR ‘Father’ OR ‘Husband’ AND ii) ‘Breast cancer’ OR ‘Breast carcinoma′ OR ‘Breast neoplasm’ OR ‘Breast tumour’ AND iii) ‘Experiences’ OR ‘Perceptions’ OR ‘Perspectives’ OR ‘Opinions’ AND iv) ‘Treatment’ OR ‘Approaches’ OR ‘Outcomes’. Multiple variations of the keywords were used including the truncations based on database requirements to broaden to capture all relevant studies.

### Inclusion and exclusion criteria

This review included all primary studies of any design (qualitative, quantitative, or mixed methods) that report on MBC (included only men assigned male gender at birth); studies focussing on the experiences, perceptions, and treatment approaches for MBC; as well as studies conducted and reported in English (based on the resources available to the researchers). However, letters, editorials, commentaries, perspectives, case reports, opinion pieces, news reports and systematic reviews on MBC; studies reporting on cancers in men other than MBC; those that did not report on MBC experiences; as well as those reported in languages other than English were excluded.

### Data extraction, quality assessment, synthesis and analysis

Search results were imported into Endnote reference manager (version 20) by the first reviewer (MA-O), duplicates removed and titles as well as abstracts were screened. The remaining studies were screened against the inclusion/ exclusion criteria, by three reviewers (MA-O, JB, OA), and any study for which inclusion was unclear was discussed and resolved by YS and TNA. Full texts studies were obtained if abstracts did not have enough information to determine the relevance of an article. Study variables such as authors, countries where studies were conducted, aims/objectives, study design, sample size and characteristics, experiences of MBC with verbatim quotes, MBC treatment approaches with outcomes and conclusions drawn were extracted to a common table (see Table 1).

**Table 1:** Data extraction on the experiences and management approaches of MBC from included studies

Authors	Country	Aim/ objectives	Study design	Sample characteristics & size	Experiences of MBC	Available verbatim quotes	MBC management approaches	Conclusion	QATSDD quality grading (%)
1. Adekolujo et al. 2017 [39]	USA	Aimed to establish the impact of MS on tumour stage at the time of diagnosis and survival in male breast cancer	Quantitative	Men with breast cancer who are with 18 years and above, diagnosed from 1990 to 2011 and with confirmed histological invasive breast cancer	N/A	N/A	Not reported	Future study on male breast cancer should verify the outcomes of this study and comprehensively test the psychosocial program of support, which is tailored to the needs of unmarried males with breast cancer in addition to the implementation	High (95)
2. Ahmed et al. 2012 [40]	Nigeria	Aimed to report the clinical and pathological characteristics and treatment in addition to outcomes of male breast cancer observed over a decade from 2001 to 2010	Quantitative	57 male breast cancer patients diagnosed between January 2001 and December 2010. The mean age was 59 with 31(54.4%) and 26(45.6%) being affected in their left and right breasts respectively. Symptoms lasted mediumly for 11 months with 28(49.1%) patients mainly presenting to the institution while 12 patients were managed by traditional healers from 7 to 36 months. 45(79%) commonly presented with breast lump, 25(43.9%) with breast ulceration and (8.8%) with nipple discharge	N/A	N/A	Surgery, Radiotherapy Chemotherapy Hormonal therapy	Educating male breast cancer patients and their healthcare providers is crucial to increasing awareness of male breast cancer and ensuring patients present early for timely referral, diagnosis and treatment. Multidisciplinary treatment approach should be adopted still following female breast cancer recognized pattern	Moderate (55)
3. Avila et al. 2023 [41]	USA, UK, Spain, Australia, Canada, Italy, South Africa, Switzerland, and Netherlands	To evaluate the presence of cancer-related symptoms and treatment side effects from the perspective of MBC patients	Quantitative	127 participants completed the survey with age ranging from 33 to 88 years (median 64). Age at diagnosis ranged from 29 to 74 years (median 55) and time since diagnosis *n* = 40 was diagnosed 1—5 years prior to their entry in the study; *n* = 50 in the previous 6 to 10 years, and *n* = 33 were more than 10 years. Participants were at different stages of the disease and from 9 different countries: USA, UK, Spain, Australia, Canada, Italy, South Africa, Switzerland, and Netherlands	*N* = 91 MBC patients reported experiencing late effects of their cancer or treatment including *n* = 71 experiencing physical symptoms (mostly fatigue); *n* = 51 had psychological effects (mostly fear of recurrence); *n* = 63 had hot flashes relating to their treatment; *n* = 69 felt less masculine as a result of their illness or treatment; *n* = 100 reported an impacted on their interest in sex; *n* = 75 were bothered about hair loss related to treatment; *n* = 70 had pain in the scar area lasting longer than usual surgery recovery; *n* = 42 had some degree of swollen arm or hand; *n* = 66 had some difficulty with their arm or shoulder movement as a result of their surgery; and *n* = 20 (15.7%) did not feel their medical team had experience in treating MBC	N/A	Mastectomy; systemic chemotherapy and endocrine therapy	Our study provides critical information on several side effects and late effects that are experienced by male patients with breast cancer. Further research is necessary to mitigate the impact of these effects and improve quality of life in men	High (79)
4. Bootsma et al. 2020 [42]	Netherlands	Aimed to evaluate the unmet information needs of healthcare providers and male breast cancer patients	Mixed methods	12 focus groups with male breast cancer patients and 2 partners. Of 107 (72%) male breast cancer patients, 77 questionnaires were completed	65% and 56% of male breast cancer patients lacked information about acute or late side effects respectively, especially sexual side effects	*Delay of diagnosis and symptoms* “After I discovered a lump, I went [to the GP] right away. First, I ended up with a replacement (for my GP) and he said it [breast cancer] wasn't possible” *Follow-up and treatment options* “Didn't you use Adjuvant online?” “For me, for instance, chemo still provided 4% better chances. I deliberately chose, precisely because of neuropathy, not to opt for chemo. I thought of the gain. Chemo can also go so wrong that you cannot even do your grocery shopping as a 45-year-old man. That's a choice, and it was made jointly”. *Coping and psychological impact* “[The diagnosis] hit me hard, emotionally”. “It hits you hard. More surgery. You just have to wait and see if [cancer] comes back”. “It's not that I go around and tell people I was in the hospital, oh, what for? What I mean is, a lot of people do not know about my surgery at all”	Not reported	Male breast cancer specific information tool in the form of a targeted website is required to improve timely diagnosis, treatment, quality of life and survival	High (95)
5. Brain et al. 2006 [43]	UK	Aimed to assess psychological distress prevalence in male breast cancer including factors that influence increased distress	Quantitative	161 male breast cancer patients with an average age of 67yrs. Majority of the participants were married or living with a partner, had at least a secondary level education with 2.9yrs mean time since diagnosis and 35% reported having a family history of breast cancer	N/A	N/A	a) Depressive symptoms and anxiety, b) Cancer-specific distress, c) Body image, d) Doping, e) Support and information needs, including f) demographic and clinical variables	About 1/4 of male breast cancer patients experienced symptoms of traumatic stress specific to breast cancer with low prevalence of depressive symptoms and clinical anxiety. Factors influencing distress included negative body image, fear and uncertainty regarding breast cancer	Moderate (61)
6. Chichura et al. 2022 [44]	USA	To assess MBC patients’ opinions and perspectives about the surgical approach for their breast cancer and to compare their experiences with surgeon recommendations for MBC	Quantitative	63 MBC patients and *n* = 438 surgeons were surveyed online. The mean age of patients was 62 ± 11 years (range 31–79 years), and the majority reported their race/ethnicity to be non-Hispanic white (*n* = 55). Most patients (*n* = 52) had been treated in the United States, with representation from the Northeast (*n* = 10), the Midwest (*n* = 9), the South (*n* = 19), and the West (*n* = 11). Of the 438 surgeons survey, *n* = 298 were female, *n* = 215 were fellowship-trained, and *n* = 244 had been practicing for 16 years or longer	N/A	N/A	Types of surgeries offered: Majority of surgeons routinely offered breast cancer support to eligible men, while others routinely offer reconstruction, and some offered reconstruction only if the patient requests it	The study found discordance between MBC patients’ satisfaction with their surgery and surgeon recommendations and experience. These data present an opportunity to optimize the MBC patient experience. We also advocate for the inclusion of men in clinical trials, the creation of trials specific for MBC, and the enrolment of patients in a prospective international registry similar to the International Programme of Breast Cancer in Men, a global effort aiming to characterize MBC biology and develop clinical trials	High (72)
7. Co et al. 2020 [7]	Hong Kong	To investigate the reasons for late diagnosis of male breast cancer	Quantitative	56 men with breast cancer, with a median onset age of 61 ranging from 33 to 95yrs, a positive family history of breast cancer and managed from January 1998 till December 2018	31 male breast cancer patients were interviewed via telephone of which 18 and 11 patients reported experiencing “very” to “extremely” severe and mild to moderate embarrassment at symptom onset respectively: only 2 having no experience of embarrassment. In addition to experiencing "extreme" or "very" severe embarrassment, 19 patients also reported prolonged clinic waiting times, they again disclosed and discussed with their spouses when they first discovered the symptoms of breast cancer with 3 and 1 patient discussing with friends and sibling respectively. Finally, 8 patients reported never talking to anyone before medical consultation	N/A	Palliative treatment- 6 Mastectomy with axillary dissection- 36 Mastectomy with sentinel node biopsy- 14	As male breast cancer is rare patients mostly delay in seeking medical attention due to lack of knowledge, public education and embarrassment hence there is the need for improved psychosocial support for patients	Low (45)
8. Crew et al. 2007 [45]	USA	Aimed to assess race and factors predicting treatment and survival of men with stage 1 to 3 breast cancer	Quantitative	510 male breast cancer patients made up of 356 whites and 34 blacks. Of these, 94% of patients had mastectomy with 28% and 29% receiving chemotherapy, and radiation therapy	N/A	N/A	Mastectomy, lumpectomy, chemotherapy, and radiotherapy	An association has been found between the black race and increased in male breast cancer specific mortality after adjusting for known clinical, demographic, and treatment factors. Future study should examine these disparities	Moderate (62)
9. Cronin et al. 2018 [46]	USA	To understand the relationship between age and male breast cancer regarding how it presents, managed, clinical outcomes in addition to other factors such as clinical, pathological as well as patient-related factors	Quantitative	152 males with breast cancer; median age reported as 64 years (range, 19–96 years)	N/A	N/A	Surgical intervention and chemotherapy with chemotherapy receipt more likely among men up to age 65 years	Men aged up to 65 years received more chemotherapy with improvements in overall survival but no breast cancer specific survival, compared with men older than 65 years	Moderate (67)
10. Donovan & Flynn 2007 [31]	UK	To elicit the lived experience of MBC patients regarding their psychosocial and psychosexual challenges	Qualitative	15 participants; 5 from UK and 10 overseas	Four major emerging themes were described: a. MBC constituting a distinctive experience with the thought of living with an illness associated with women causing distress and stigma among men b. Also, there was an overwhelming change in the notion of their embodiment, constituting a substantial change to their body image and sexuality, reinforcing the experience of erectile dysfunction among men with tamoxifen therapy c. Unfortunately, some care providers could not provide psychosocial support resulting in marginalization regarding the possible effects of the environment of treatment d. Nevertheless, there were some men who adjusted through reassert and renegotiation of masculinity as they found opportunities of accommodating life with the stigma and the alteration in their body image	*Making Sense of Male Breast Cancer: “*(Doctor) said to me it was Estrogen amenable. I assumed it was caused by an excess of estrogen in me which is a female hormone. (Doctor) told me that it is a female gene that I’ve got in me” *The Context of Masculinity: “*This has killed my sex life; I can no longer get an erection. I’m on this Tamoxifen which I’ve got to take for 5 years You know it’s driving me mad. I got free Viagra but there is nothing there. There’s no feelings or anything like that and it’s terrible. I don’t know what it was, I just felt (silence) I just felt so embarrassed.” *The Stigma of Male Breast Cancer: “*I want to prove to everybody that male breast cancer is not a women’s disease and that a normal man can have it”	Not reported	The experiences of male breast cancer depict a contestation of masculinity and the legitimacy of owning the disease. Nonetheless, men adopt and adapt characteristics of masculinity such as patience, self-determination, and courage to overcome these challenges. Care providers have the chance of offering possible endorsement of renewing the masculinity of men with breast cancer instead of upholding possible emasculation	High (86)
11. Duarte et al. 2017 [47]	Brazil	Aimed at knowing the context being sick and surviving breast cancer among men	Qualitative	Two men (66 & 74yrs) who survived breast cancer. A 74-year-old was widowed, childless, retired, Catholic, did not complete elementary school and was diagnosed in 2007. A 66-year-old, married with three children, retired, a farmer, protestant, did not complete elementary school and was diagnosed in 2007	After the diagnosis of cancer, men managed to lead a normal life with limitations and changes in daily life, including suspension of work. It was perceived that optimism, and the acceptance of the disease were fundamental to face and adapt to these adversities. In relation to coping and survival, while one of them resorted to denial as a way of dealing with this situation, the other sought acceptance. Regarding support network, family and friends contributed to obtaining positive effects in the treatment of one participant	*The discovery of breast cancer*: “No, I never felt anything, I was shaving in a big mirror and when I saw blood came out through my breast. Funny. Did it break a vein? I thought, and that's when I went to get medical help. But I did not imagine it was cancer, because I did not know about it" *Coping with a breast cancer survivor*: “You have to accept what it is like I always thought positive, always forward, you do not have to warm your head I feel calm like this, I was never nervous about it, until today everything is normal. No good thinking bad things, I just thought of good things” *Sources of support for men surviving breast cancer*: “My sister who was always by my side, together, she always accompanied me. They (children) always stayed by my side giving me support. At the time I needed them, they helped me (friends)”	Radiotherapy, chemotherapy & surgery	This study created awareness about the context of men when getting sick and surviving breast cancer, as it allowed to observe the steps that involve the process of discovery, treatments, coping, survival, daily life, and support networks	Moderate (69)
12. El-Beshbeshi & Abo-Elnaga 2012 [48]	Egypt	To report clinicopathological characteristics, treatment patterns, and outcomes of men with breast cancer	Quantitative	37 men with breast cancer with a median age of 57.7yrs ranging from 26 to 86yrs. 94.5% of these men reported a mass under their areola with local advancement and their tumors were invasive duct carcinomas	N/A	N/A	Treatment was mainly surgery in 91.8%, followed by adjuvant 89.2% radiotherapy, 56.7% hormonal and 91.8% chemotherapy	Male breast cancer is most often diagnosed in an advanced stage making the management of male and female breast carcinoma is identical	High (76)
13. France et al. 2000 [49]	UK	To describe the psychological and social consequences of the diagnosis of breast cancer in men	Qualitative	6 male breast cancer patients who completed a radiotherapy/ chemotherapy course with accompanying spouses being invited for comments as appropriate	The 7 themes that emerged included: a. “delay in diagnosis” b. “shock”, c. “stigma”, d. “body image”, “causal factors”, “provision of information” and “emotional support”	*Delay in diagnosis***:** "Noticed the lump in April, went to the GP in August about something else. The doctor was convinced it was nothing to worry about, but I pushed the point that I did have private medical care. If I hadn’t pushed the point, he would have left it at that juncture" *Reaction to diagnosis*: "I found it totally shattering. Then the Consultant suggested referral for a mammary strip and to a Consultant Oncologist and Radiotherapist, by this time of course I thought my last days had come. I said, ‘At the worst what is the prognosis?’, with this he said ‘at the worst you’ll be dead in 12 months so I thought I had better put my house in order” *Stigma*: "No embarrassment, the mates don’t actually understand, they don’t ask you". *Body image*: "Of course it doesn’t matter to a bloke, but I wouldn’t go swimming anymore. I could tell a very good tale about how I was in the Hussars or something and get away with it. I am very conscious about it (the scar), I wouldn’t display my chest to the boys or my grandchildren". *Causal factors*: "I made a few trips to Abercomboi to where the Furnacite plant was I worked on the coal, but dust and nothing bothered me". *Provision of information*: "I wasn’t given any literature, but my friend Audrey was given a lot of literature, and she gave me several leaflets. I got more or less the idea, but you feel a bit of a ninny when you’re reading all this about putting your bra on and that sort of thing" *Emotional support and counselling*: "They don’t cater for men. There is a programme coming up in Cancer Awareness week, it’s all for women you know I feel like writing to say that men can get breast cancer you know."	Radiotherapy and/ or chemotherapy	There are psychological and social issues for men with breast cancer, which impact on their management and care. It has been recommended that developing a structured education program for all primary care providers including pre and postoperative gender-specific information that can minimize the potential psychological issues that come with the diagnosis. Additionally, appropriate counselling/ support services should be provided for partners of male breast cancer patients	Moderate (52)
14. Giordano et al. 2005 [50]	USA	To describe the experience of institution-wide adjuvant systemic therapies in male breast cancer	Quantitative	There were 135 nonmetastatic male breast cancer with age ranging from 25 to 80yrs and a median age of 59yrs at diagnosis. Pre-dominantly there were 72% white, 15% black and 12% who were Hispanic	Not reported	N/A	Surgical with adjuvant therapy and chemotherapy with radiation, and hormonal therapies	Men who received adjuvant hormonal therapy experienced a significant overall survival of 0.45; *P* = 0.01 suggesting the benefit men derive from adjuvant therapies with a scale similar to that seen in women	High (93)
15. Halbach et al. 2020 [51]	Germany	To explore the healthcare situations of men with breast cancer from their perspectives	Mixed methods	27 males with breast cancer	Before diagnosis, men with breast cancer reported seeing their primary care physician when either they or their partners observed indicators like lumps, bleeding in the nipple or breast pain. Others reported lack of expression of suspicions on the part of primary care physicians regarding the indicators being signs of breast cancer as they were referred to physicians not providing care for breast cancer, or they were told to observe it for a while. These issues led to delays and late diagnosis with some diagnosis happening months and years after the initial indications were seen. During treatment, there were expression of satisfaction with some men feeling safe and well informed by providers. Side effects were liked with Tamoxifen treatments including sexual dysfunctions, sweating, memory loss, hot flushes, sleep disorders and joint pain among others. During rehabilitation, men reported experiencing being alone among women with some feeling isolated and excluded. Some reported a lack of aftercare guidance although others experienced trust, regular and comprehensive guidance regarding breast self-examination	“Well, my primary care physician already suspected that it could be breast cancer, and therefore, first mammography. Then the women were split up in all these other rooms and I suddenly had a four-bed room for myself. They made an insane effort there no one could tell me what the side effects were of tamoxifen. This person, it’s a man, but it’s just an affected person. At the water aerobics, I was also the only one. Because they said they do not want, that the women with breast cancer, that there is a, a man. Because some women may not want that, yes”. I say: “OKAY”, I say: “So I’m alone in the swimming pool.”	Not reported	There is the need to increase male breast cancer awareness among researchers, healthcare workers and the public so as to prevent late diagnosis, reducing stigmatization and indecisions around its management. treatment. Also issues around access to care and aftercare guidance should be addressed	High (83)
16. Harlan et al. 2010 [52]	USA	Aimed to assess the features, management, and survival among newly diagnosed men with breast cancer between 2003 and 2004	Quantitative	Men with first diagnosis of breast cancer at the age of 20 from January to December 2003 *Sample size* 512 randomly selected men from the SEER database from each participating registry *Age distribution* < 50:59 (11.5%) 50–59:118 (23%) 60–69:136 (26.7%) 70–79:115 (22.5%) > = 80:84 (16.4%) *Racial distribution* non-Hispanic white- 400 non- Hispanic Black- 61 Hispanic- 32 Asian- 19 *Cancer staging* Insitu: 58 Stage 1–3: 392 Stage 4: 36 *Unknown staging*: 26	Men who were not currently married received chemotherapy significantly less often and had higher Cancer mortality than married men	N/A	*Surgery and Radiation* No surgery Breast conserving surgery with radiation Breast conserving surgery without radiation Mastectomy and radiation Mastectomy without radiation *Chemotherapy and Hormone therapy* No adjuvant therapy Chemotherapy only Chemotherapy + hormone therapy Hormone therapy only	The primary predictors of mortality and therapy among men with breast cancer were marital status and tumor features. There is the need for future research to assess the association of gonadotropin releasing hormone analogues with the effect of aromatase inhibitors	High (74)
17. Hill et al. 2005 [53]	USA	Aimed at using Surveillance, Epidemiology, and End Results data (SEER) in describing male breast cancer epidemiology in comparison with gender and race-specific incidence trends in determining the association between breast cancer disease-specific survival and demographic or tumor features	Quantitative	There were 2923 male breast cancer recorded on the cancer registries participating in the SEER betweeen1973 and 1999 with an average age of 64.8 at diagnosis *Staging n(%)* In situ 157 (11.6) Localized 622 (46.1) Regional 486 (36.0) Distant 85 (6.3) *Race n(%)* White 2,449 (84.6%) Black 323 (11.2%) American Indian 5 (0.2%) and 117 (4.0%) Asian/ Pacific Islanders	The risk of breast cancer death is 21% higher for those who were not currently married	N/A	Not reported	After adjusting for demographic variables, gender was not a significant predictor of survival although some important gender differences were detected with respect to factors associated with tumor features. A large-scale analysis of gender-specific survival, with treatment variables and demographic factors in the current study is recommended for future research	Moderate (69)
18. Hiltrop et al. 2021 [54]	Germany	To explore the experiences of men with breast cancer as they ‘return to work’ (RTW) using an explorative qualitative approach to determine: (a) the kind of existing RTW patterns (b) the motivation to RTW; (c) the experiences of RTW and (d) the effect of male breast cancer on work after RTW?	Qualitative	*N* = 14 out of *n* = 27 interviews were analysed with a total of 100 men with breast cancer participating of an average age of 66.9 yrs and a subsample of 14 participants having an average age of 58.6 yrs. Those interviewed were first diagnosed 4yrs prior to the study with *n* = 8 working full or part-time and *n* = 6 were retired or on sick leave with all having varying levels of education during data collection	The description of RTW patterns focused on: a. ‘working during therapy’, b. ‘participation in medical rehabilitation’, c. ‘occurrence and type of RTW’, and d. ‘job changes after RTW’. Of the 14 interviewed, 11 patterns were analysed with more than one patient experiencing patterns 5 and 6, with four patterns indicating participants working while on chemotherapy and /or radiation treatment. These waived the option to RTW slowly. Changes were reported after RTW including reducing hours of work, different tasks, retirement, and taking on new roles	*Handling cancer disease in the workplace*—"And in my life, I have generally gotten into the habit of going on the offensive right away and putting all my cards on the table. This is because nothing is more boring than yesterday's rumour. If you try to fiddle or cover things up, they will keep asking: ‘Well, what do you have? What's that? And why isn't he showing up now?’ So, I wrote an email and took the big distribution list, everyone I could think of and sent it off." "After the reintegration, you're suddenly back in working life. It's like turning a switch. You simply have to function again. Your colleagues quickly forget that you were gone for eleven months, not long ago. Expect a lot of understanding but offer little themselves. You always have to show understanding for them and their situation, always." *Changes in productivity after RTW*—"And the first workday would have been the same day as my first follow-up appointment, right? But I already told my boss: ‘I can't come in then, that's when I have my follow-up appointment,’ right?" "In the beginning, oncology had actually wanted physical therapy five times a week. And now because I also travel for work, I do three times a week. And I simply don't have time for more either."	Surgery Chemotherapy Radiation therapy Hormone therapy	Decisions in relation to RTW are taken in different healthcare contexts requiring various opportunities for supporting male breast cancer survivors influencing their RTW patterns and rates. For Germany, there is a provision of 3 weeks medical rehab for patients within the health system allowing for gradual RTW options and measures of support which enables participating in work life	High (73)
19. Hoffman et al. 2020 [55]	Israel	Aimed at presenting an overview of the outcomes and experiences of men with breast cancer in Israel covering over 22 years in addition to reviewing clinical and oncological outcome changes over time	Quantitative	Men with breast cancer who had surgery from January 1993 to December 2015 *Sample size*: 49 with an average age of 64.1. There were Ashkenazi Jews: 66% (*n* = 29); Sephardic Jews: 22.7% (*n* = 10); non-Jews: 12.2% (*n* = 6) Unknown: 9% (*n* = 4)	N/A	N/A	Mastectomy + Sentinel Lymph node biopsy + Level 1 Mastectomy + Axillary Lymph node dissection Radical mastectomy Radiotherapy Hormonal therapy	Male breast cancer is a rare disease that continues to increase. Negative status of PR has been linked with better overall survival and disease-free interval	Moderate (50)
20. Iredale et al. 2006 [13]	UK	Aimed at investigating the experiences of male breast cancer throughout the UK	Mixed methods	Phase 1 is a focus group discussion with *n* = 27 participants made up of two groups of male breast cancer (*n* = 5 & *n* = 4), one group of female breast cancer (*n* = 13) and one group of care providers (*n* = 5) Phase 2 is a survey with *n* = 161 male breast cancer participants. Phase 3 is interviews with *n* = 30 men from phase 2 Phase 4 is reconvening of focus groups consisting of *n* = 7 male breast cancer and *n* = 10 female breast cancer participants	**Qualitative** a. Many men were rather shocked at the receipt of breast cancer diagnosis as this was seen to be a female disease b. Information shared with participants were through leaflets, booklets, verbal and by internet sources and photos prior to surgery although most of this information were female related c. Formal support services were underutilized with few participants speaking to other men with breast cancer although some would have wished to have such support post diagnosis d. Most people just do not accept the possibility of men being diagnosed with breast cancer. Participants felt the need to raise awareness on male breast cancer among care providers as well as the public especially regarding symptoms of the breasts or nipples **Quantitative** Most men disclosed their diagnosis to spouses/ partners (*n* = 129, 80%) and other close family and friends, with less disclosure to extended family and work colleagues (*n* = 60, 37%). A small number of men (*n* = 6, 4%) disclosed to no one The most common source of information for participants was verbal (*n* = 148, 92%), with 71% (*n* = 114) receiving leaflets and 53% (*n* = 85) receiving booklets; in addition, 20% (*n* = 32) had used the internet, while 12% (*n* = 19) saw a photograph prior to their surgery. Information was primarily delivered by healthcare professionals working in hospital settings, but much of what was available in written form was inappropriate, covering topics such as menstruation, breast reconstruction, and bra fittings. Over half of participants (*n* = 90, 56%) wanted much more information Demonstrated low utilisation of formal support services. Only 19% of participants (*n* = 31) spoke to other men who had breast cancer, but 27% (*n* = 43) would have liked that opportunity after their diagnosis. Most were not interested in talking to other men or women with breast or other cancers either individually or in a group and the vast majority would not attend a gender mixed support group	*Diagnosis and disclosure* ‘Now when I first knew that I had got it, I thought to myself well how the Dickens did I get breast cancer. I’m not a woman. I’m a man’’. ‘‘I was surprised more than anything. Women it’s an ever-present threat. Men never occurs to them’’ *Information needs* ‘‘No information. Nothing at all. I daresay women aren’t left like that. On leaving after the first operation the nurse gave me a leaflet with women on it doing exercises you have to do and that was it’’ *Support* ‘‘My wife was my support she and I talked about everything. At the beginning we talked about it and agreed that I would have her as my support and she would have her family to support her through. It worked well and I also got support from her family, mine were useless’’. ‘‘None of the guys wanted to have self-help groups I don’t think they need the psychological support that perhaps women do. I think this is, of course research I know but actually quite therapeutic in a way’’ *Raising awareness* ‘‘By their expression they don’t believe me. You can tell they think I am conning them you know, lying to them or whatever’. ‘‘Yes they were incredulous and then a couple of them laughed’. ‘‘I guess every article you ever read is about women with breast cancer. And nothing ever says oh by the way chaps you can get it too I don’t think raising awareness about it would be difficult it would just be about including men’’	Hormone therapy, mastectomy, lumpectomy, chemotherapy and Radiotherapy,	Findings show that men have valuable and constructive things to say about how their breast cancer care should be delivered if given the opportunity to share their experiences. There is therefore a need for future research with lager sample of men with breast cancer, exploring their experiences throughout the disease trajectory with its corresponding management	Moderate (62)
21. Kowalski et al. 2012 [56]	Germany	Aimed at describing health related quality of life among German men with breast cancer and to explore any significant differences among male and female	Quantitative	There were *n* = 84 men with breast cancer of an average age of 64.82	N/A	N/A	84.5% had mastectomy; 7.1% had either breast-conserving therapy or partial mastectomy, and 8.3% did not indicate the type of surgery	There was a significant health related quality of life score of 7 out of the 8 sub-scale for men with breast cancer compared to that of women with breast cancer	High (86)
22. Levin-Dagan and Baum 2021 [20]	Israel	To explore ways men cope with the threat of being stigmatised as a result of being diagnosed with what is perceived as a woman’s disease	Qualitative	*N* = 16 men ranging from 25 to 78 years (median = 59) and diagnosed with breast cancer within the past 10 years as well as communicated in Hebrew were interviewed with a mean interview time of 51 min (range 33–75)	Reported as verbatim quotes	*Being treated in a female-patient-oriented healthcare system*—'I received an instruction page written in the female gender with instructions to sleep with a bra and all sorts of things that are connected only with women, not with men. But I don’t know if there are any special instructions for men. They just don’t know, it’s like that.' *Reactions to being a man diagnosed with breast cancer—'*… even now in the radiation treatments, there are male nurses who take you inside and prepare you, and after 20 or so treatments we kind of bond, and finally you’re lying on the table and he touches and moves you, and says: “Tell me, bro, may I ask, how did you discover it? I’ve never heard of this before.” And I said to myself, wait a minute, you work here and you see things, so that means that it really is uncommon. *Selective disclosure*—[My friends] still don’t know that it’s breast cancer. Because it still somehow makes me feel ashamed … It’s still awkward. It’s inconceivable in here [pointing to his head], it doesn’t make sense. It’s as if a woman said she had prostate cancer. I don’t know. It’s contradictory. *Body concealment—'*I feel different in that I have only one breast. So I do my best not to take my shirt off because it makes me feel bad to be different. Look, if it’s at the Dead Sea, who else is going there? Only those who have all kinds of things. Even there I didn’t take it off, I went in the water with my shirt on because I felt ashamed. It’s not really shame. I don’t know … My feeling is that people won’t talk, there you are, all eyes are turning toward me and see that I have only one breast. There are people who don’t give a damn, but regarding this, I do	Mastectomy, chemotherapy, radiation therapy, and hormonal treatment	The study reveals MBC patients manage their discrediting position of being diagnosed with a “woman’s disease.” Our findings add to the understanding of the stigmatisation experience and address for the first time men’s coping mechanisms	High (75)
23. Miao et al. 2011 [25]	Denmark, Finland, Geneva, Norway, Singapore, and Sweden	Aimed at comparing incidence trends with relative survival and excess mortality among male and female breast cancer to understand outcomes and risks in men relating it with women breast cancer	Quantitative	Participants with breast cancer diagnosed between 1970 and 2007 except Denmark, where inclusion was up to diagnosis in 2006. A total of 2665 was included representing 677 for Denmark, 347 for Finland, 61 for Geneva, 435 for Norway, 74 for Singapore and 1,071 for Sweden with 69 as the median age	N/A	N/A	*Treatment* Surgery n (%) Yes 728 (86.4) No 79 (9.4) Unknown 36 (4.3) *Radiotherapy* Yes 251 (29.8) No 447 (53.0) Unknown 145 (17.2) *Chemotherapy* Yes 127 (15.1) No 542 (64.3) Unknown 174 (20.6) *Hormonal* therapy Yes 190 (22.5) No 508 (60.3) Unknown 145 (17.2) Total 843 (100)	Over the last four decades, the risk of male breast cancer continues to persist. Generally, men with breast cancer have worse survival rates but when adjusted for life expectancy, year of diagnosis, age, treatment and stage of disease, and male patients with breast cancer emerged as having a survival benefit compared with women	High (86)
24. Midding et al. 2018 [32]	Germany	Aimed at examining the feelings of men with breast cancer regarding suffering from a “woman’s disease”	Mixed methods	Qualitative interviews were conducted with *n* = 27 men with breast cancer in addition to *n* = 100 quantitative data collected using questionnaires	**Qualitative** Five main categories of stigmatization were identified: a. *Experience of stigmatization* describing scenarios of men with breast cancer being treated differently compared to other patients b. *Bodily dimension* including facets that are linked to changes occurring in and to the body as well as the body image after surgery c. *Indirect stigmatization* comprising scenarios causing shame and indisposition leading to self-stigmatization **Quantitative** there is significantly less stigmatization with close family and friends than in broader social settings, for instance, with colleagues Most stigmatization takes place in rehabilitation settings (mean = 1.50), significantly more than during chemotherapy (*p* = 0.006), radiation (*p* = 0.019), follow-up survey (*p* = 0.031), and within family (*p* = 0.004)) In the cancer care system, the men experienced significantly higher stigmatization during hospitalization (mean = 1.20) than during chemotherapy (mean = 1.14; *p* = 0.049). The experienced stigmatization is higher within the cancer care system than within social surroundings	*Experience of stigmatization* “I remember that woman in the breast cancer center. She said: ‘What do you want here? You don’t belong here.’ “I think I was called ‘Mrs. Miller’ once. Something like this is also unpleasant.” *Bodily dimension* “This is a time when the disease is also disfiguring. Nobody sees the surgery. You have your scars, but you can hide them. But when the hair is gone, mustache away, eyebrows away.”; “[While sitting in the waiting room] the women are thinking: ‘He accompanies his wife. She’s in treatment.’ And when you’re being called: ‘Mr. Miller please.’ All heads are turning, and you feel kind of observed.”	Surgery Chemotherapy Radiation therapy Antihormone therapy	Findings suggest that men with breast cancer experience stigmas mostly within the care system, therefore management strategies should be developed for it. There should also be male breast cancer awareness creation to provide equality cancer care to ensure that breast cancer is seen as a disease for both men and women	High (79)
25. Midding et al. 2019 [57]	Germany	Aimed at exploring: (a) resources for social support (b) types of support being used, and (c) to identify the heterogeneity of the resources used by men with breast cancer	Mixed methods	127 participants; 27 qualitative interviews 100 questionnaire with patients with MBC Participants were 66.9 years on average and only 30.9% were still working (full-time and part-time)	MBC patients received three supports: a. emotional support, usually from their informal caregivers b. informational support from health professionals. Support needs are dependent on factors such as the level of disease, age, patient’s education, copying style c. Instrumental support. Cancer support groups provide both informational and emotional support **Quantitative** Workplace Colleague support: social support resource of colleagues was not available for most participants Private peer support: most men (63.2%) have contact with female Breast Cancer Patient (73.1%) for support. In comparison, 24.2% of the participants have contact with other MBCP Group peer support: 15.3% of the participants are part of a support group; the majority (84.7%) of participants are not. Most participants who were not part of a support group stated that they do not wish to be part of a support group (96.3%) Described 3 broad typologies of social support use among male breast cancer patients Type 1: does not use any social support during the breast cancer dis- ease. But the Modified Medical Outcomes Study Social Sup- port Survey short scale (mMOS-SS) identifies that this group mostly have someone who offers them emotional (mean = 4.4) and instrumental support (mean = 4.5). Average age of 78yrs Type 2: Majority of MBC patients fall within this group. They use different resources of social support from one to three categories of social support (emotional, informational and instrumental) during the process of disease. They use a minimum of two resources. The total score of the social support scale indicates that they mostly have someone who offers them social support, but the mean value of support received is the lowest among the groups (mean emotional support = 4.2, mean instrumental support = 4.4). They have a younger average age (66.6 years) compared to Type 1, Type 3: receives social support from two or all three categories of social support. This type uses the most diverse resources of support and has the highest amount of used support. The availability of social support has the highest mean value of the types (mean emotional support = 4.7, mean instrumental support = 4.8). They are the youngest type with an average age of 57.5 years	*Need of support from support group:* “I had no interest in that I said: Okay I had it, but it’s over Basically, I don’t want to always be confronted with it they partly described their complaints there.” *Emotional support*: “My wife is also the first contact person for me, of course.” *Informational support:* “My sister is a doctor. That’s also my best guide. She isn’t a medical specialist. She’s an anesthesiologist, but of course has contacts. And of course, then can enlighten directly.” *Instrumental support*: Wife: “He can’t wash himself properly. So, I washed him. I also put some cream at him at the moment, I cut his fingernails and toenails.”	Chemotherapy, Adjuvant radiation, Hormone therapy	Depending on the age, occupation and severity of male breast cancer, the identification and usage of social support could differ. As older men with breast cancer whose disease is less severe use less social support and vice versa. Partners of men with breast cancer and closer social environment are key resources for inclusion within the cancer care system. Future research should assess the use of healthcare professionals as a resource of support for male breast cancer	High (81)
26. Nahleh et al. 2007 [58]	USA	Aimed at comparing the outcomes and features of male and female breast cancers	Quantitative	612 male breast cancer has been compared with 2413 female breast cancer. The average age for male and female breast cancer at diagnosis was 67 and 57 years respectively. Majority of male breast cancer were black with ductal carcinoma dominating the histology and presenting in an advanced stage	N/A	N/A	There were 36% of female breast cancer receiving chemotherapy and 34% radiotherapy compared to 29% and 20% of male breast cancer respectively. However, both male and female breast cancer equally received endocrine hormone therapy	Findings indicate that variations exists in terms of pathology, biology, presentation, survival and the ethnicity of male and female breast cancer	High (86)
27. Nguyen et al. 2020 [34]	Germany	Aimed at investigating male breast cancer experiences in order to ascertain their support and care needs	Qualitative	18 men with breast cancer aged between 53–83 yrs; and mean time of diagnosis being 4.5yrs ranging from 2 to 8 yrs	The participants expressed different views regarding receiving a diagnosis of a “women’s disease.” a. While some participants thought of male breast cancer as unusual and threatening their manliness, others thought of it as “any other disease” b. The experience of stigma was highlighted which threatened their sense of masculinity c. With regards to their body image, the scars from the surgery didn’t seem to bother participants though some confess to hiding it initially. Although loosing hair as a result of chemotherapy became a huge worry for some participants. d. Regarding treatment, the male participants reiterated that approaches to managing the disease were designed mainly for women resulting in men feeling prevented from getting satisfactory care. The men voiced their desire at exploring the effect hormonotherapy had on them as they reported effects of hot flashes or sweat with some men considering themselves to be “menopausal women" due to the side effects. Despite these concerns, they were generally satisfied with their care e. With regards to coping, participants who are married reported mostly receiving social and emotional support from their spouses in helping them to cope. Additionally, some confess that it was their wives that edged them on to seek medical help that resulted in the breast cancer diagnosis. They also found support groups helpful	a. Living with a “Women’s Disease”: “My biggest problem was how to tell my wife that I have a woman’s disease? Because I thought maybe you’re not a real man, perhaps half woman?” i. *Stigma* “From others at work, I always (hear) ‘admit it, you’re just trying to find excuses. You’re not a real man, or you wouldn’t have such an illness’.” ii. Body Image “It’s a different situation for women; in your mind it’s then more about losing your femininity and who knows what else. But that’s not the case for us, you see? I’ve only got one nipple left, right? That doesn’t bother me” b. Barriers: *Hormonotherapy* “I would have quite liked the anti-hormone therapy to include a medication that had been tested on men, so that I could be confident that it’s suitable for me, as a man.” c. Coping: i. *Wives’ Support* “I’m glad I have my wife I don’t know how it would have ended.” ii. Support Groups “To be honest, I don’t know how I would be managing if I had never had (the support group). They gave me back the will to live and I will always be grateful for that.” d. *Supportive Care Needs*: “Social and psychological support could be strengthened right at the beginning, when you get the diagnosis.”	Use of pharmacological agents and surgical intervention (mastectomy)	It is crucial raising awareness on male breast cancer including adapting management approaches with adequate information for patients and available support services aimed at improving male breast cancer care	Moderate (64)
28. Özkurt et al. 2018 [59]	Turkey	Aimed at studying the clinicopathology outcomes and features of male breast cancer in emphasizing the variations in comparison to female breast cancer	Quantitative	53 male patients diagnosed with breast cancer, underwent surgical operation attended routine follow-up from January 1993 to April 2016; Median age 64 (34–85) with all participants at different stages of disease ranging from 0 to stage 3	N/A	N/A	*Type of surgery n(%)*: 1 (2) breast conservation; 11 (21) mastectomy and 41 (77) underwent modified radical mastectomy *Sentinel Lymph Node Biopsy n(%)* Yes 20 (37.7) No 33 (62.3) *Axillary Lymph Node Dissection n(%)* Yes 42 (79.2) No 11 (20.8) *Systemic treatment n(%)* 7(13.2) underwent preoperative systemic chemotherapy; 25(47.2) had chemotherapy and 21(39.6) had no treatment 32(60.4) had radiotherapy and 21(39.6) did not; 45 (90) had hormonal therapy and 5(10) did not	When compared to female breast cancer, male breast cancer had a different clinicopathological and prognostic factors. Hormonal therapy has become the main management for estrogen receptor male breast cancer due to the high rates of hormone receptor positivity	Low (47)
29. Potter et al. 2023 [60]	USA	To better understand how men experience changes in occupation when diagnosed with breast cancer	Qualitative	*N* = 24 MBC participated in the interview lasting 10 to 100 min. The average age of the participants was 57.75 years, the median age was 59 years, and the mode was 70 years of age	Most participants found meaning in new occupations related to advocacy. This new role centred around building awareness and support for men past, present, and future with breast cancer through public speaking, joining MBC organizations, participating in research, volunteering, and educating others. Some participants expressed the move into advocacy as a natural progression from their profession. Men described themselves in terms of becoming activists. Multiple participants started nonprofit foundations focused on meeting individual and societal needs related to male breast cancer	*Social Environment*—So, I found the men’s group. And even though I was the only one with breast cancer, I found it more comfortable and helpful. But I think that fact they had a specific men’s group, it was very important in my journey and in my recovery…I really found that women would react to me in a different way (P-23). *Decrease in Occupational Engagement due to Side Effects*—I was off for a couple weeks for my surgery and then when I was going through chemotherapy, I would take off the day of the chemotherapy initially and then towards the end of my chemotherapy um started to catch up with me. So, I was working four days a week when I began my chemotherapy and by the end, I was working three days. (P-15) *Finding Meaning in New Occupations*—The goal was if I can help one male through that, it was done. I achieved my goal. But one person, you know, I mean once you get that satisfaction from that, you just have to keep going. (P-16)	No specific treatment was reported in the data	Occupational therapy has the ability promote engagement in meaningful occupations and therefore promote overall health and well-being in the lives of men affected by breast cancer through understanding the unique barriers and successes men in this study described. The men in this study expressed instances where they did not feel welcome in the healthcare environment and their health care providers were not well versed in treatment of male breast cancer. Only through a client-centred and occupation-based approach will occupational therapy benefit clients to achieve optimal occupational engagement	High (76)
30. Rayne et al. 2017 [61]	South Africa	Aimed at describing and assessing the perceptions of men with breast cancer regarding their manliness and the effect of having a disease that is commonly attributed to women	Quantitative	There were 23 men with breast cancer involved at various stages of the disease and non-metastatic at diagnosis	Of the *n* = 23 participants, only *n* = 6 had knowledge of male breast cancer before they were diagnosed and only *n* = 1 was keen to disclose the disease and its management to their relations. Participants did not agree that breast cancer influenced their insight regarding their manliness except *n* = 5 that thought otherwise. Black participants and those managed in state hospitals were unlikely to have knowledge on male breast cancer but could more likely have their perceptions regarding their manliness affected Only five (17%) respondents noted feeling embarrassed about taking off shirt in public now All but one patient willingly disclosed their disease and treatment to their family and friends	N/A	Surgery, chemotherapy and radiation if needed	Most of the participants’ perception of their manliness and relationships were not affected by being linked to a female disease, although black participants and those receiving care in state hospitals reported differences. The likelihood of these links having substantial influence on some worried men with breast cancer is pertinent to supporting them especially those receiving care in state institutions	High (86)
31. Sanguinetti et al. 2016 [62]	Italy	Aimed at evaluating the clinicopathological features, biology and genetical impacts including the management and outcomes of male breast cancer	Quantitative	47 men with breast cancer with a mean age of 67yrs with diagnosis being established in an average time of 16 months of symptoms onset and sub areolar swelling being the key clinical complaint	N/A	N/A	Radical mastectomy; Modified radical mastectomy; and Lumpectomy. All patients received adjuvant therapy following surgery; Radiation therapy; Hormone therapy, and Chemotherapy	The prognosis of the MBC is undoubtedly worse of breast cancer in women	High (71)
32. Sarmiento et al. 2020 [63]	USA	Aimed at comprehensively describing male breast cancer tumor and clinical features and to explore factors affecting survival	Quantitative	There were 16,498 men with breast cancer having medial age of t63yrs. Over 75% of men presenting with breast lesion were found to be malignant with an invasive ductal carcinoma	N/A	N/A	Primary resection surgery was commonly used; Lymphadenectomy; hormonal therapy was also commonly used. Chemotherapy was used and radiation therapy in patients	As found in female breast cancer, there was a significant association between surgery and improved survival in men with breast cancer. Factors noted for affecting male breast cancer survival include increase in age, being black, access to state insurance, multimorbidity and tumor stage being high	High (79)
33. Shah et al. 2012 [64]	India	To analyse the clinicopathological profile of men with breast cancer	Quantitative	*N* = 42 men with breast cancer having average age of 56yrs ranging from 31 to 78yrs and various stages of cancer diagnosis	N/A	N/A	Most men with breast cancer had surgery at different stages of the disease, some had modified radical mastectomy and radical mastectomies. Men who had surgery were also given chemotherapy as well as radiotherapy. Those who had failed tamoxifen were given chemotherapy and the rest had both chemotherapy and palliative radiotherapy where there was metastatic bone	The majority of MBC are found to be hormone receptor positive, hence hormonal therapy should be strongly considered	High (79)
34. Shin et al. 2014 [65]	USA	Aimed at comparing overall survival and racial variations in the management of men with breast cancer	Quantitative	There were 4,279 men with breast cancer of with 3,266 being White, 552 Black, 246 Hispanic, and 215 Asian	N/A	N/A	Not reported	Overall, the findings indicate that Blacks are disadvantaged in comparison with Whites, Hispanics, and Asians in relation to survival have a survival, which could partly result from variations in the way the disease presents. Additionally, it was discovered that lymph node dissection, which could be beneficial to patients were less likely to be received by Blacks after stratifying for disease stage as an underlying factor which contributes to the disparities in survival outcome. Future research is required to explore whether racial disparities in men with breast cancer have any association with access to care, socioeconomic status, genetics and biologic etiologies as well as limiting medical comorbidities	High (90)
35. Sineshaw et al. 2015 [18]	USA	Aimed at examining the extent of the differences in the receipt of treatment and survival for black or white men with breast cancer at an early stage	Quantitative	There were 5,972 men comprising 725 blacks and 5,247 whites of age 18 and above, whose diagnosis ranged from stage 1 to 3 between 2004 to 2011. *Age distribution* 18–39 159 (2.7%) 40–54 1308 (21.9%) 55–64 1607 (26.9%) 65–69 874 (14.6%) 70–79 1351 (22.6%) > = 80 673 (11.3%) *Cancer staging* Stage 1—2549 (42.7%) Stage 2—2523 (42.2%) Stage 3—900 (15.1%)	N/A	N/A	*Definitive* locoregional therapy including surgery and radiotherapy *Adjuvant* hormonal therapy *Adjuvant* chemotherapy	Although black and white men have similar rates of treatment receipt at an early stage of breast cancer, the risk of death is higher among black men of age18 to 64yrs however this is not the case for those aged 65yrs onwards, who have moderate Medicare insurance coverage. These findings suggest the importance of ensuring that men with breast cancer have access to care in order to reduce ethnic/ cultural differences in mortality among men with breast cancer	Moderate (69)
36. Skop et al. 2018 [66]	Canada	Aimed to understand the experiences of men who had mutation of BRCA and were screened for breast cancer	Qualitative	15 men with breast cancer with an average age of 55 ranging from 40 to 76. They were all Caucasian with children. *N* = 13 were married and *n* = 2 divorced with most of the being Jewish or Catholic and working as teachers, health professionals owning business, as well as *n* = 4 retired	Findings: Body appearance is important (“Guys don’t have breasts.”), for example, using the word chest as opposed to breast “chest” rather than breasts	Themes emerged include: a. *Body talk*—"If I talk to my friends, and I haven’t because I don’t have cancer, but if I did I would talk about it as chest cancer. I wouldn’t use breast cancer. So that would be the term I would use and, in the conversation, I would say that it is the same as breast cancer. It’s exactly the same thing; it’s just it’s in my chest". b. *Changing awareness of breasts:* "I was getting a little soft in the area I joke with my son and say “they’re my moobs” my man boobs right because the pectoral muscles, if you don’t stay on it and keep them firm, they start to look a little more like breast I don’t really think about [chest] too much other than that fact I probably should do some toning" (laughs) c. *Experiences of undergoing mammography* "I wandered into the waiting area, the breast exam area and sat down there with the women and when they came out they asked for Mrs. Smith, Mrs. This, Mr. and s o I stood up and there were a couple of giggles and titters and then we wandered in and they separated the women to one side and I went to the other"	Mammograms	The findings of this study showed that there is limited research male breast cancer in association with masculinity, which has the potential to lead in the improvement of men with breast cancer’s BRCA experience in addition to improvement in they are screened	High (73)
37. Thompson and Haydock 2020 [67]	USA	To listen to men’s breast cancer career stories with the guiding question “What are men’s dis/embodied experiences as they journeyed their breast cancer career?” Looking beyond the expected assault-disruption story	Qualitative	17 MBC patients were interviewed from different locations within the USA: *n* = 7 from New England, *n* = 3 each from Southern and Western states, *n* = 2 each from the Midwest, and East coast states. Their age ranged from 37 to 82 All the men were partnered/married at diagnosis and were predominately White (*n* = 15), with one each African American and Native American. All but three men (82%) had gone beyond a high school education; six (35%) had earned post-bachelor’s degrees	16 out of the 17 men reported being surprised and did not believe their diagnosis of breast cancer, which demonstrates the near invisibility of MBC. Two overarching themes were identified within their narrative experiences*: body talk* and *embodiment of their breast*. Men’s body talk centered on their discovery of having breasts, the implications of their surgical wound and hormonally unruly bodies, and living with a cancer-injured body, with narratives equally conferring how they came to embody having breasts. Renegotiated embodiment describes navigating foreign (women’s) spaces, telling others about their breast cancer, and reformulating their subjective masculinities	a. Body talk—*Having breasts*: “I didn’t even want to think that I have breasts let alone have a cancer in my breast.” *Surgical wound*: “I didn’t have a shirt on; I do that on the beach. You know, after the surgery there’s obviously a scar there and no hair, you know, cause of the radiation. But I don’t care, you know.” *Unruly bodies*: “First of all my hair fell out because of the chemo, and I immediately cut the rest of it off. I told the people at work that I just liked to have a bald head.” b. Embodiment of their breast—*Navigating foreign spaces*: “We’re men in this pink world and it’s uncomfortable. So you read some of the websites, you read some of the brochures that are available in the clinics and um, you know, you have a hard time even knowing that this is a disease that men can get.” *To tell or not*: “Socially, I don’t bring it up. But obviously everybody knows, my circle of friends, everybody knows.” *Reformulating masculinities*: “For the first several months I was wary about not wearing a shirt. Now, on the beach I didn’t have a shirt on; I do that on the beach. You know, after the surgery there’s obviously a scar there and no hair, you know, cause of the radiation. But I don’t feel a concern, you know.”	Mastectomy	The men shared narratives on men’s agency and their widening of traditional masculinities. MBC patients volunteered to provide testimony at breast cancer events, urging clinics and their physicians to give men the opportunity to mentor new MBC patients, or pushed pink ribbon organisations to be more inclusive and include men as “poster boys” in breast cancer calendars or for newspaper articles	High (71)
38. Visram et al. 2010 [68]	Canada	To examine the rates of adherence to and toxicity from endocrine treatments in male breast cancer patients treated at a single institution	Quantitative	There were *n* = 40 men with breast cancer in their early and advanced stages with a median age of 68 ranging from 46 to 84 at the Ottawa Hospital Cancer Centre from 1981 to 2003	N/A	N/A	a. Primary surgery b. Adjuvant radiation therapy c. Adjuvant chemotherapy d. Hormonal therapy	The study found toxicity association with endocrine therapy in male breast cancer as reported in female breast cancer This imply that men are are as likely to stop their endocrine therapy as early as did women with breast cancer	Moderate (57)
39. Wang et al. 2019 [69]	China	Aimed at analysing and comparing clinicopathological features, incidence trends, and survival outcomes in male and female breast cancer	Quantitative	2,254 men with breast cancer and 390,539 women with breast cancer were involved with median age of 65yrs and 59yrs for men and women respectively. Compared with	N/A	N/A	Mastectomy, radiation, and chemotherapy	Findings show that clinicopathological features, biological behavior and clinical outcomes in early male breast cancer varies from that of female breast cancer	Moderate (53)
40. Weber et al. 2021 [70]	Germany	(1) To describe defensive functioning, repressive coping, and fear of progression in a sample of male breast cancer patients, (2) To describe patterns of defensive functioning in relationship to repressive coping in male breast cancer patients, and (3) To explore the possible impact of repressive coping on an association between fear of progression and defensive functioning in male breast cancer patients	Quantitative	Participants were recruited nationwide through certified breast cancer centres, members of the MBC network, and invitations through newspaper advertisements with a median age of 60 years (ranging from 39-89 years). All participants had a confirmed breast cancer diagnosis for the first time, although the time window since diagnosis varied averaging just under 4 years. *N* = 100 men completed the quantitative survey, and a subsample of *n* = 27 took part in the qualitative interviews according to purposeful sampling	Male breast cancer males have a mean Overall Defensive Functioning (ODF) value of 5.62 (SD = 0.82) with 30% exhibiting mature defense organization (e.g., superior healthy neurotic functioning); 26.9% showing immature defense patterns regularly found in patients with personality disorders (e.g., borderline) and depressive disorders while majority of the sample showed neurotic defense patterns 46.2% of the sample (*N* = 12) used a non-repressing coping strategy versus 53.8% (*N* = 14) repressors. Both groups did not differ in age, marital status or disease duration. Use of non-repressing coping styles was associated with previous experience with breast cancer in the family (X^2^[1, *N* = 26] *r* = 5.60, *p* < 0.05) There was higher use of mature defense patterns (superior healthy neurotic functioning) in patients who use non-repressive coping ODF was significantly associated with fear of progression (*r* = 0.43, *p* < 0.05) i.e. the higher the fear of a worsening of cancer, the higher levels of (more adaptive) defensive functioning. Also under conditions of no repressive coping, higher levels of fear of progression were associated with higher levels of (more adaptive) defensive functioning	N/A	Surgery, chemotherapy, and radiation therapy	MBC patients are co-treated in a more feminine setting specializing in treating women with breast cancer leading to the experience of stigma. Therefore, consideration of coping with the disease including a more conscious coping strategies and a more unconscious defence mechanisms appears to be very helpful for MBC patients to recognize the distress and neediness, which may be hidden behind gender models. A better knowledge of the specific disease management could be followed by interventions, as early as possible and prospective controlled studies are needed for this purpose	High (71)
41. Williams et al. 2003 [71]	UK	Aimed at identifying issues of importance in helping men cope with breast cancer	Qualitative	Total sample of 27 also involving female breast cancer and care providers Unclear how many of the 27 were male breast cancer	a. Prompt diagnosis as wife prompted and nudged patients to report symptoms to healthcare professional b. Men reacting stoically to reception of diagnosis c. Concern about disclosure d. Concerns about appearance- some concerned and others not e. Lack of peer support groups of males with shared lived experiences f. Objection to mixed gender support groups g. Lack of tailored gender-specific breast cancer information for men h. Lack of representation within breast cancer information e.g. pictures of men with breast cancer and their surgery scars	‘She [wife] said ‘You’ve got to go. I’ve made an appointment” ‘I don’t discuss it openly with anybody unless it is directed at me’ ‘I’ve been abroad and sunbathed. People do look, they do look. People don’t care. Only you care. Nobody else cares. After a while you get to know that. They just look at you and say ‘Oh’ ‘One of the worst things was the fact there weren’t any men I could go to’ ‘I think a male photography that way you are showing someone this is what is going to be you after the operation’	N/A	The findings of this study is a confirmation that there is limited male breast cancer specific information for them to access especially for those who may have specific issues of concern relating to their appearance after being diagnosed and managed for breast cancer	Moderate (55)
42. Yadav et al. 2020 [72]	India	To analyse outcome in MBC patients with adjuvant treatment	Quantitative	81 MBC were retrospectively analyzed for patient‐related characteristics such as age, comorbidity, family history, pathological stage/tumor size, histology, grade, extracapsular extension, lymphovascular invasion, estrogen/ progesterone, and treatment‐related factors such as radiotherapy, chemotherapy, and hormonal therapy	N/A	N/A	Adjuvant hypo fractionated radiotherapy received by *n* = 51, chemotherapy by *n* = 35, and tamoxifen by *n* = 45 men with breast cancer	The adjuvant treatments used resulted in significant improvement on disease‐free survival and overall survival in men with breast cancer except chemotherapy, which had zero effect on disease‐free survival and overall survival	Moderate (76)
43. Yoney et al. 2009 [73]	Turkey	To evaluate the general features, treatments applied, and the results obtained in male breast cancer patients	Quantitative	*N* = 39 men with breast cancer made up of 94.8% invasive ductal, 2.6% invasive papillary and 2.6% invasive lobular carcinomas distributed according to stages 1 (12.8%); 2 (46.2%); 3 (30.7%) and 4 (10.3%) of the disease	N/A	N/A	Patients received radiotherapy and hormonotherapy, chemotherapy, chemoradiotherapy and others received hormonotherapy in addition to surgery	As a way of improving local control, use of radiotherapy postoperatively was significant in the treatment men with breast cancer	High (71)
44. Zongo et al. 2018 [74]	Burkina Faso	Aimed at studying the diagnostic stage, modality for therapy and 5-year survival for men with breast cancer	Quantitative	51 men with breast cancer representing 2.6% of all men whose diagnosis happened around the same time. Their median age was 60.9 yrs with age groups from 61 to 70yrs being the most represented	N/A	N/A	Surgery followed by chemotherapy, radiation and hormonal therapy	Male breast cancer diagnosis remains late. The most basic management approach is surgery. The choice of molecules and the number of cures, as a result of the cost of cytotoxic has limited the use of chemotherapy. The 5-year survival set out remains slow with median survival depending on the stage of diagnosis Increasing awareness campaigns including the organization of screening for individuals might reduce late diagnosis thereby improving recovery	Moderate (62)

**Fig. 1:**
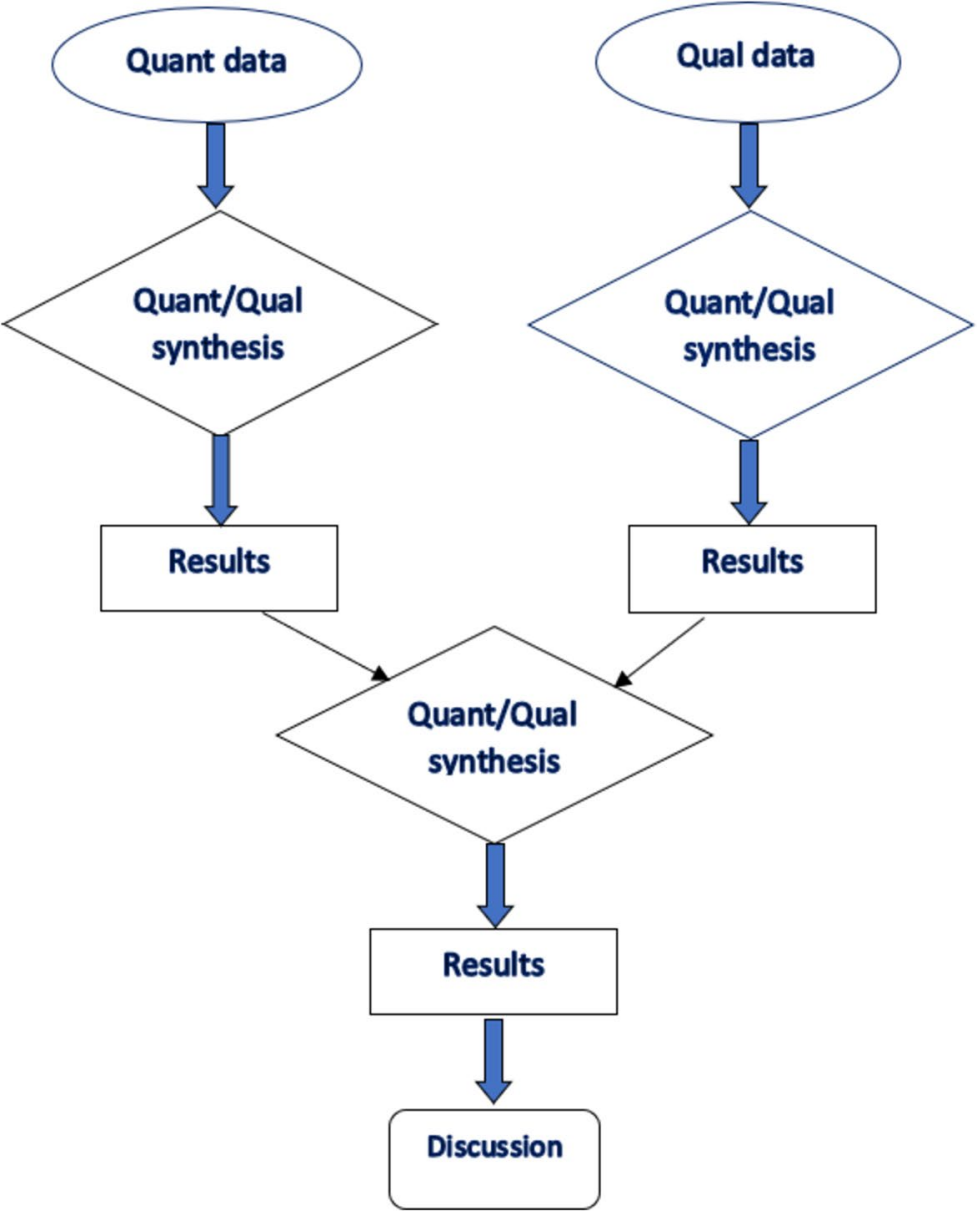
A flow diagram on the results-based convergent design

We used a results-based convergent design [75] to guide data analysis, where we initially synthesised qualitative and quantitative findings separately, before integrating these findings from the two designs in the final analysis and synthesis (see Fig. 1). This allowed us to synthesise quantitative findings regarding treatment approaches of MBC and qualitative or mixed methods results on the experiences of MBC patients.

**Fig. 2:**
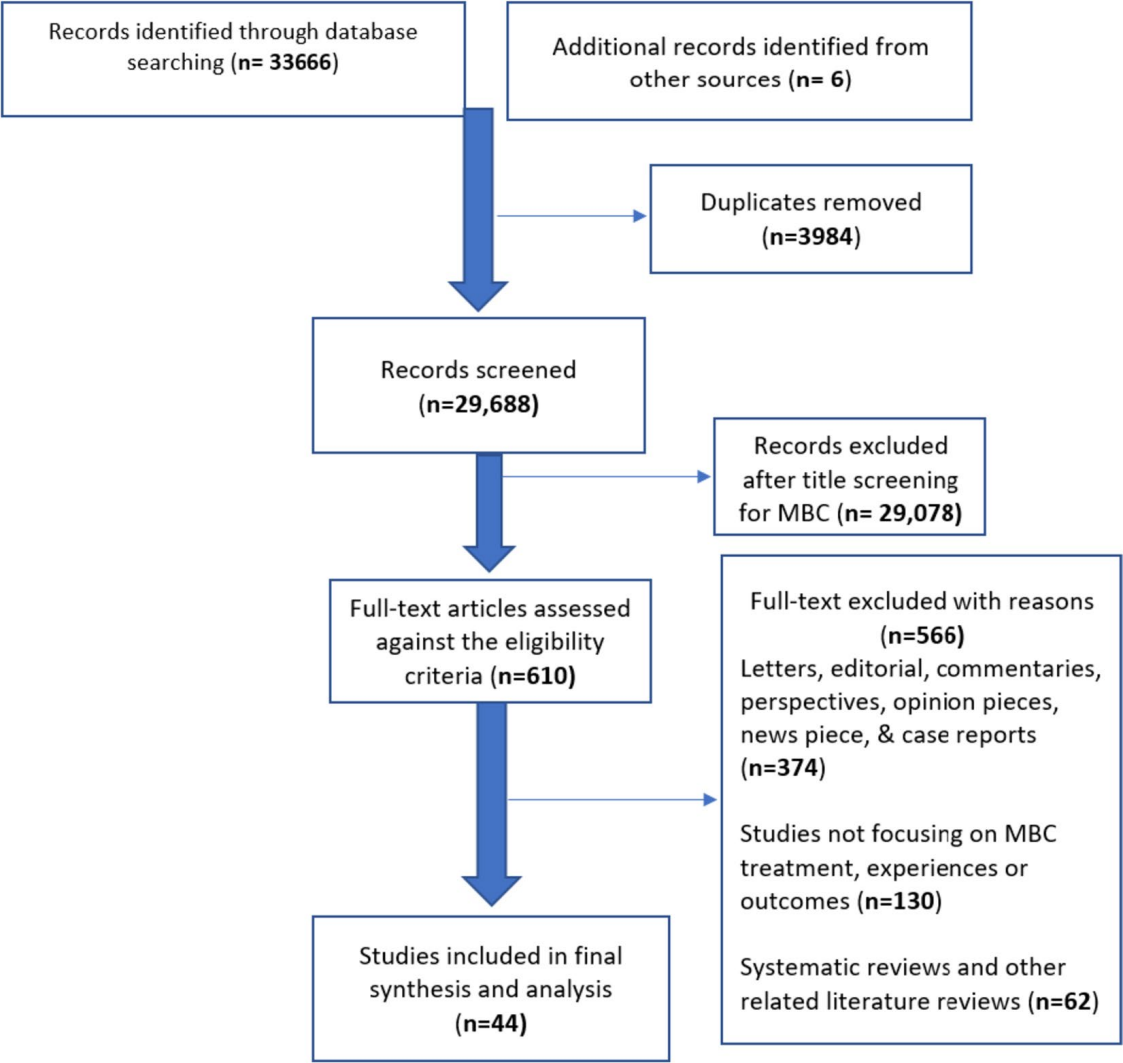
PRIMA flow chart of study search and selection process

Descriptive statistics was used in reporting the number of published studies and presented in a PRISMA flow diagram in Fig. 2. We synthesised the descriptions of MBC experiences and treatment approaches reported across studies. All studies were analysed descriptively. To synthesise the data regarding the experiences of men with breast cancer, verbatim quotes reported in the qualitative studies were extracted by two authors (JB & TNA). An interpretive and inductive stance was employed [37] by reviewing verbatim quotes to generate codes (see Table 2). Similar codes were aggregated to generate sub-themes followed by formulation of higher order themes. For the quantitative data regarding the treatment modalities, we focused on describing the main reported treatment modalities rather than their frequencies. At the end of the analysis, both the qualitative findings and descriptions from the quantitative studies converged as one dataset. The themes generated from the initial process and the descriptions obtained from the quantitative studies formed the basis of undertaking a narrative synthesis.

**Table 2:** Themes, sub-themes and codes

Themes	Sub-themes	Codes
Navigating through a threat to masculinity and one’s existence	1. Emergence and awareness of a ‘foreign’ illness and threat to one’s existence 2. Coming to terms with a gendered disease 3. Opening up/ coming out of the illness closet	● Living with an exotic or women’s disease
		● Threat to oneself and coming face to face with one’s mortality/ death
		● Shock and needing emotional support at the cancer diagnosis
		● Feeling like an outsider/ stigmatised as being the only male patient with breast cancer at the hospital/ gynaecological units
		● Until diagnosis, most men did not know about MBC (which delayed the timing of seeking healthcare/ diagnosis)
		● Left in the cold after receiving diagnosis of cancer
Navigating through treatment	1. Therapeutic interventions 2. Navigating through feminised treatment pathways 3. Living with the effects of care/ ongoing treatment	● Hair loss from chemotherapy
		● Mastectomy scars
		● Breast cancer treatment (and some procedures) was developed with women in mind. So, taking the same hormonal therapy medications such as Tamoxifen as females and its subsequent side effects such as hot flushes, sweating and decreased libido/ lowered sexual potency were considered frustrated ● Being treated as a woman going through treatment pathways designed for women ● Men with breast cancer felt health care practitioners did not know much about their disease and treatment regimen; some practitioners lacked sensitivity and did not take the patients seriously ● Difficulties with finding a doctor to treat them due to reimbursement issues (the GPs felt their specialty was women’s practice and did want to attend to the men with breast cancer) ● Generally, they were satisfied with the medical care; some felt the services and procedures at the hospital failed to consider their needs) ● Some men were unconsciously addressed as ‘Mrs’ in waiting rooms or in their letters ● General lack of male-specific psychosocial support and information tailored to their needs ● Some men or their wives had to persist before being referred to the consultant surgeon ● Living with mastectomy scars/ body image changes ● Feeling that exposure to environmental toxins had caused the cancer ● Questioning about the cause of the cancer ● Lack of information specifically about breast cancer ● Being put on medication originally prescribed for females with breast cancer (Tamoxifen) ● Postoperative support and advice were lacking ● Not surprised to be diagnosed with breast cancer, but the men were shocked at receiving a diagnosis of breast cancer as it is considered a gendered disease ● Some men disclosed to close family and friends and others did not disclose to anyone ● Not wanting sympathy or to be stigmatised ● Lack of awareness as perceived about breast cancer among men ● Feeling awkward while discussing sensitive issues
		● Wishing their condition were called something else, rather than breast cancer ● Younger men affected by altered body image than older men ● General lack of information about breast cancer and the treatment process in males ● Most were generally not interested in talking to other men with other forms of cancers ● Participants did not describe delay in seeking healthcare ● Wives/ partners played a key role in pushing for early health seeking ● Men reacted stoically following breast cancer diagnosis ● Healthcare professionals were less sensitive and “matter of fact” attitude ● Not fully open about their diagnosis ● Some men were concerned about their appearance (some would stare at their scars; unable to remove their shirts during outdoor events) ● Disappointed at the lack of information on breast cancer specific to men ● Men showed pictures of females who had undergone mastectomy and not male mastectomy ● Majority of men would appreciate a chance to discuss with another man with breast cancer on basis ● Receiving the cancer diagnosis as a lightning strike ● Being scheduled for mammography or being told of having a lump led to feelings of being men with breasts ● Feeling dumbfounded with a cancer diagnosis and its location ● Feeling of having breasts, not only a chest ● Feeling like a freak because of the gendered status of the disease ● Living with visually disturbing mastectomy scars ● Concerns about the wound, but not the so significant gendered part ● Men concerned about their body image and upper-body mobility following affecting mastectomy ● Mastectomy re-sculptured their muscles necessitating a need to amend their masculinity ● Living with a disfigured chest from the scars ● Living with the side effects of the adjunct hormone therapy (sudden mood alterations, hot flushes, emotional explosions, PMS, altered sexual lives, loss of erections etc.) ● Younger men more concerned about their physical bodies than older men ● Managing breasts and masculinities ● Having troubles with scheduling mammography (feeling like the only male in the sorority) ● Embarrassing to interact with healthcare staff about MBC ● Feeling out of place/ alone at clinics ● Lonely process in coming to terms with the reality of the diagnosis/ having breasts ● Being the only man among women during rehabilitative care (some experienced positive experiences as they got the chance to share with the women) ● Feeling exotic and excluded from the group during the rehabilitative/ aftercare phase ● Not seen as gendered malignancies like testicular cancer which participants could lay a legitimate claim of ownership ● Giving the illness a gendered status ● Associating the illness more closely with femininity than masculinity ● Body image changes/ sexuality concerns ● Some surgical procedures following MBC impacted on their masculinity, and in some instances, their sexual orientation ● Public information regarding male breast cancer is scarce ● Distress because of living with inaccurate information on the disease and misunderstandings ● The experience of loss of libido and erectile dysfunction following tamoxifen therapy. This impacted on their masculinity ● Healthcare staff were excellent but were often unaware of the specific information and psychological needs of men ● Feelings of being marginalised in the clinics (as HCPs attempted to conceal them from the female clients by asking them to wait in other parts of the clinic or use alternate entry/ exit routes)
Coping and support systems	1. Active coping strategies 2. Family support 3. Support from healthcare providers and other support groups	● Healthcare staff were excellent but were often unaware of the specific information and psychological needs of men
		● Some men experienced reluctance in sharing their unusual problem or disclosing their problem
		● Trying to find the right name for the disease (chest cancer, cancer on the chest etc.)
		● Support from wives, and family
		● Receiving emotional support from wives, partners, and other female friends
		● Attending support groups (although others were sceptical about joining)
		● Other coping strategies included physical activity, acupuncture, psychosocial services at the hospital
		● Unwilling to discuss MBC diagnosis with other family members/ close friends; but they did not feel embarrassed
		● Need to speak to men with similar experience of MBC
		● Although dealing with the disease had been difficult, younger men reported gaining new insights in life and changing their views and life priorities
		● Amending or reformulating their masculinities (men with breasts and cancer, seeking emotional support from close friends and partners, opening up to others about their cancer experiences)
		● Concealment a life-threatening cancer and its location to manage their sense of oddity
		● Feeling lucky of having the cancer at a part not considered “vital”
		● Mixed reactions of the physicians (some referred immediately, and others did not express suspicion about a cancer diagnosis/ wait and see attitude which led to late delays with diagnosis)
		● Difficulties in accessing gynaecological care (rejection by some healthcare centres due to billing issues)
		● Some men were satisfied with in patient care and did not differ from routine care; some men felt being in a special position such as receiving more attention from healthcare professionals whilst others did not feel comfortable with it as being the only male in a room for a procedure whereas the women were divided into the rooms

The quality of included studies was assessed using the Quality Assessment Tool for Studies with Diverse Designs (QATSDD) tool [76], which is designed for use in mixed methods reviews and quality reporting in reviews that included qualitative, quantitative, mixed- and multi-methods research to ensure consistent and critical appraisal of relevant studies. In assessing study quality, studies were categorised as high quality if they achieved an aggregate score in excess of 70%, moderate quality were assigned to studies scoring between 50 and 70%, and those scoring less than 50% were assigned low quality (see Table 1). However, no study was excluded based on respective aggregate quality scores.

## Results

### Study characteristics

Of the *n *= 610 full-text articles assessed for eligibility. *N* = 374 were excluded as these were letters, editorials, commentaries, perspectives, case reports, opinion pieces and news reports on MBC; including *n* = 130 studies that did not report on MBC experiences and perceptions; and *n* = 62 that were MBC related reviews (see Fig. 2). Following extensive search and screening, 44 studies were retained in the final synthesis and analysis, with publication years ranging from January 2000 to September 2023. Twenty-nine studies employed varied quantitative designs, 8 studies employed qualitative designs, and 6 studies employed mixed-method designs. Although most of the studies (n = 44) included only MBC, two retrospective studies compared males and females with breast cancer, and only the data reported on males were included in this review [58, 68]. Study characteristics including quality assessment grading are reported in Table 1.

### Experiences and perceptions of males with breast cancer

As shown in Table 2, three themes and nine sub themes emerged from the data which encapsulate the experiences of males with breast cancer.

#### Theme 1: Navigating through a threat to masculinity and one’s existence

This theme describes how males experienced the illness reflecting on detection, diagnosis, coming to terms with the disease, and disclosure. The subthemes are 1) emergence and awareness of a foreign illness and threat to one’s existence 2) coming to terms with a gendered disease and 3) opening up/ coming out of the illness closet. All included nine qualitative studies highlighted how the affected men perceived breast cancer as a threat to their sense of masculinity.

### Emergence and awareness of a foreign illness and threat to one’s existence

Males generally perceived breast cancer as a feminine illness which cannot affect their bodies [31, 34]. In fact, although all the men in the included studies had heard about breast cancer, most of them had not previously heard about breast cancer in males which made them rule out any possibility of ever living with it and may have contributed to delay in seeking healthcare [31, 49]. This perception and the emerging non-specific symptoms often delayed early health seeking as the symptoms were interpreted as irrelevant or not requiring urgent attention [49]. It is worth highlighting that most of the affected men presented with palpable lump in the breast or discharge from the nipple of the affected breast. Some men had to be ‘pushed’ by their wives or partners to seek medical attention to rule out the possibility of breast cancer; a condition they felt was out of their scope [49, 71]. A breast cancer diagnosis was met with varied emotions including being dumbfounded, shocked, surprised, debilitating stress, and a feeling of housing a feminised illness in a masculine body which threatened their sense of masculinity and personhood [13, 31, 34, 49].

*“…there is no reason why I shouldn’t have cancer, I’m only the same as anyone else. I’m just a bit disappointed really about where it got me. it’s not right on a man, is it? *[31]* (p.467).*

*“From others at work, I always (hear) ‘admit it, you’re just trying to find excuses. You’re not a real man, or you wouldn’t have such an illness’. *[34]* (p.8).*

*‘I suppose the fact that it was breast cancer surprised me. The fact that it was cancer I suppose was a shock . . . So, I suppose a combination of both. You know the fact that it was breast cancer which I do not think I had heard of and the fact that it was cancer’’* [13]* (p.336).*

Receiving the diagnosis was challenging which some men kept to themselves or only informed family/ close friends [71]. The notion of breast cancer being a feminine illness made men view the disease as foreign or exotic to their bodies [49]. The growing awareness of the disease made the men feel a sense of oddity and shame for having a feminine illness alongside a feeling of losing one’s manhood to an illness not considered masculine [31, 49]. Worry, anxiety, and uncertainty also marked their increasing awareness of the disease particularly regarding how the disease could distort the shape of their ‘masculine chest’ [13]. Despite the varied emotions, some males felt extremely lucky that the cancer was located at a site not considered ‘vital’ in terms of masculinity [67].

*My biggest problem was how to tell my wife that I have a woman’s disease? Because I thought maybe you’re not a real man, perhaps half woman?” *[34]* (p.8).*

*“Now when I first knew that I had it, I thought to myself …well how did Dickens get breast cancer? I’m not a woman. I’m a man.* I was surprised more than anything… Women, it's an ever-present threat … Men – never occur to them. ‘‘When I first knew I did not want everyone knowing, because I did not want everyone coming round sympathising’’*. *[13]* (p.336).*

Further to the above, the diagnosis of breast cancer forced the affected men to come face to face with their own mortality. This is because they felt a diagnosis of breast cancer threatened their existence and equated to a death sentence. The realisation of death lurking close by pushed the affected men to increase their efforts in attaining their dream before they died. This experience helped them to be more appreciative of their present lives, increased their consciousness about their health, and helped them to redefine their values and beliefs [60]:

“I appreciate life a lot more. Before my cancer, I didn’t take life seriously. I took life for granted. I didn’t appreciate the people in my life and the things I see. So, after the cancer, it was a good kick in the butt. Just how much you appreciate it, and also made me realise to go after my dreams, chase it, and achieve it. Go after it and every day is a gift” [60] (p.3).

### Coming to terms with a gendered disease

Through the journey of receiving a breast cancer diagnosis and living with the illness, the affected men expressed the insights and perceptions they gained regarding living with an uncommon illness that is believed to affect mostly women [60]. Following the breast cancer diagnosis, males were faced with the reality of living with a condition they did not expect to have. Coming to terms with a feminised disease was gradual and a lonely journey for the affected men. In fact, some wished they could give their condition another name instead of breast cancer. The fear of being stigmatised made some men keep their diagnosis to themselves [13, 32]. Others also felt a sense of awkwardness discussing such sensitive issues and would avoid [13]. Taken together, men with breast cancer often concealed or attempted to re-label their diagnosis to manage their sense of stigma, shame, and oddity as they navigated through coming to terms with living with a “feminine disease” in their masculine bodies [13, 32, 66]:

*‘‘I told the guys I played golf with that I’d got cancer; I do not think so. I necessarily told them it was breast cancer’’. *[13]* (p.337)*

*“…but if I did, I would talk about it as chest cancer. I wouldn’t use breast cancer. So that would be the term I would use, and, in the conversation, I would say that it is the same as breast cancer. It’s exactly the same thing; it’s just in my chest.” *[66]* (p. 964).*

*“I think among old men they almost consider it to be a stigma, they almost don’t want to tell people, you know, it’s some kind of, I don’t know, a black mark, but I never looked at it that way…I think people younger would just view it a little differently, you know it’s cancer, it’s something they have to deal with, it doesn’t really matter what type of cancer it is.” *[67]* (p.37)*

### Opening up/ coming out of the illness closet

As the men gradually came to terms with living with the “foreign or exotic disease”, they were able to talk to their families and close friends about their diagnosis [13]. This required a lot of courage to navigate through such a sensitive issue. Interestingly, the men noted that the process of openly discussing their diagnosis in social spheres and coming out to others offered them an opportunity to reassert the meaning of masculinity, particularly as they recognize how fragile their masculine bodies are [31]:

*“When I spoke to people about it, they thought I was telling fairy tales … that was really the worst thing about it.” *[34]* (p.8).*

*“I want to prove to everybody that MBC is not a women’s disease and that a normal man can have MBC.” *[31]* (p.468).*

In two studies, however, the authors described the phenomenon of selective disclosure in which the men only disclosed their illness to selected persons only [20, 60]. For some men, the selective disclosure also meant revealing just the diagnosis, but not going further to reveal how they are experiencing the treatment process or the aftermath of the illness:

“The children know and our closest friends know, the very closest. Why? Because I disappeared for a while. I don’t talk about it within the family, not at all. Nobody talks with me about it, but they know. It is only information, and that’s it, not about the experience and not about the surgery, and not about the treatment” [20] (p.5).

#### Theme 2: Navigating through treatment

The theme captures the experiences of undergoing breast cancer treatment/ management following their diagnosis. The subthemes are 1) therapeutic interventions 2) navigating through feminised treatment pathways and 3) living with the effects of care/ ongoing treatment. All included qualitative, quantitative, and mixed method studies (*n* = 44) highlighted the treatment experiences and pathways respectively.

### Therapeutic interventions

Several therapeutic interventions/ treatments were reported across the included studies. Five categories of treatments were ascertained across the included studies, and these are surgery, radiotherapy, chemotherapy, hormonal therapy, and palliative care. Surgical interventions included mastectomy with axillary dissection, mastectomy with sentinel node biopsy (both for men with late-stage breast cancer presentation), and lumpectomy [7, 40, 45–48]. Cronin et al., [46] noted that surgery and chemotherapy receipt were more likely among men up to age 65. In some studies, surgical interventions were the main forms of treatment with radiotherapy, chemotherapy, and hormone therapy playing adjuvant roles. For instance, in one study that included 37 men with breast cancer, radiotherapy (89.2%), hormonal therapy (56.7%), and chemotherapy (91.8%) were adjuvant therapies after surgery [48]. In one study, the authors reported several therapeutic regimens offered to men with breast cancer which included breast conserving surgeries, unilateral/ bilateral mastectomy, often with no reconstruction [44]. One third of the male breast cancer patients in the same study (*n* = 21) felt somewhat or very uncomfortable with their appearance after the surgery. Receipt of treatment was remarkably similar between blacks and whites in both age groups. Older black and white men had lower receipt of chemotherapy (39.2% and 42.0%, respectively) compared with younger patients (76.7% and 79.3%, respectively). Younger black men had a 76% higher risk of death than younger white men after adjustment for clinical factors only (HR, 1.76; 95% CI, 1.11 to 2.78), but this difference significantly diminished after subsequent adjustment for insurance and income (HR, 1.37; 95% CI, 0.83 to 2.24). In those age 65 years, the excess risk of death in blacks versus whites was nonsignificant and not affected by adjustment for covariates.

### Navigating through feminised treatment pathways

Despite the reality of breast cancer among males, the care pathways and healthcare payment frameworks across various healthcare systems are significantly tailored to the needs of females which reinforces the notion of the disease as a feminine in nature [31, 71]. A study from Germany highlighted the difficulty that these men experience in finding a physician as the practitioners felt their breast care specialty targeted women and would lose on reimbursement [34]. Even in facilities where they were given satisfactory care, the men felt the services and procedures still failed to consider their unique needs as men with breast cancer [31, 42, 71]. Some men were mistakenly addressed as females on the assumption that only females experienced breast cancer [34]. Male-specific psychosocial support and information were generally lacking across the studies. Information leaflets mostly contained pictures of female breast cancer patients which made the men feel excluded [34]. In fact, they felt the service was not designed for them:

*“My GP said: ‘I don’t know what to do any more, it’s not my specialty area. I’ll have to refer you to someone else’. And the other doctor said, ‘This is a women’s practice (…) and we can’t get reimbursed for men, we don’t want men here.’” *[34]* (p.9).*

*‘‘. . . but I think as a male the information that I was given was female orientated and it could have been better presented for me and . . .I know that every case is different, but it was lacking in that respect’’. *[13]* (p.336).*

Further to the above, some men had several challenges in scheduling for therapeutic regimen such as mammography [67]. Interactions with healthcare providers were often considered awkward as the providers often did not know what to say to the men with breast cancer. Subsequently, most men with breast cancer undergoing treatment often felt like outsiders, out of place, marginalised, and alone:

*‘No information. Nothing at all. It was like men; you are on your own. I daresay women aren’t left like that . . .On leaving after the first operation the nurse gave me a leaflet, a piece of paper with women on it doing exercises you have to do and that was it’’. *[13]* (p.336).*

*“I find that dealing with the mammograms and the technical staff to kind of tiptoe around you and put you in certain places because they don’t expect a male to be there, right, so they got women walking around in their gowns, so they don’t want you in those areas… they kind of shunt you into an isolated, a more isolated area so you’re not seeing the women walking by.” *[66]* (p.967).*

### Living with the effects of care/ ongoing treatment

Men undergoing treatment for breast cancer felt their lives, roles, and occupations were impacted adversely by the treatment regimen [60]. The clinical management process of the disease, in fact, further heightened the gendered essence of the disease. For men who underwent surgical intervention, the mastectomy scar served as a permanent reminder of the disease impacted on their masculinity [66]. Others felt their chest had deformed due to the scar [71]. The typical exposure of the male chest at leisure activities such as the beach was considered a no-go area to conceal the scar from public view. The scars also evoked a sense of perceived stigma among these men [32]:

*“I’ve been abroad and sunbathed. People do look, they do look” *[71]* (p.1835).*

*“I don’t feel like a complete person either because I’ve got something missing, haven’t I? ... My nipples are not there anymore. Sometimes I look in the mirror . . . I don’t like doing that. It’s gone. . . There’s a scar across there. . .Doctor said I look like a patchwork quilt. So, I don’t bother taking my shirt off now. And something else … yes you ought to have a tattoo as a nipple’’. *[13]* (p.337).*

For men who underwent hormone therapy, it was observed that the side effects of the various medications threatened their notion of being a male. Experiencing erectile dysfunction and loss of libido were really challenging for these men as they felt they had lost their sense of masculinity or what made them men [34, 77]. Hair loss from chemotherapy was also challenging and frustrating for them [43]. These men felt as though they had been transformed to ‘menopausal women’ [34].

*“We’re candid and honest with one another … male sexual potency has gone.” *[34]* (p.9).*

*“This has killed my sex life; I can no longer get an erection. I’m on this Tamoxifen which I’ve got to take for 5 years. You know it’s driving me mad. I get free Viagra but there is nothing there. There are no feelings or anything like that and it’s terrible. I couldn’t get an erection or nothing. I don’t know what it was, I just felt so no, no (silence) I just felt so embarrassed.” *[31]* (p.467).*

Further to the above, some men felt they were a burden to others as they had to rely on others to have their needs met. Younger males felt their traditional roles as providers of the family was threatened as their dependence increased with a slow return to work and had to be supported by their spouses [54]:

*“You start to receive only sickness benefits and when all of a sudden, you have over 500 euro less, you have to first see how you manage with that. And for me [...] it was even more because I only have a 60% part-time job and work as a freelancer on the side. And that I couldn't do any longer either.” *[54]* (p.6).*

#### Theme 3: Coping and support systems

The theme describes how men with breast cancer coped with the disease, treatment process, aftercare/ rehabilitative care, and the available support and it was reported across qualitative (*n* = 9), quantitative (*n* = 5) and mixed methods (*n* = 4) studies. The subthemes are 1) active coping strategies 2) family support and 3) support from healthcare providers and other support groups.

### Active coping strategies

Although the breast cancer diagnosis was considered threatening with intense emotional stress, some affected men remained optimistic and hopeful of improved outcomes. Affected men often worked towards accepting the disease which made the navigation process less challenging [47]. The treatment process and aftercare phase offered the affected men an opportunity to amend or reformulate their notion of masculinity [66]. Although dealing with the disease was difficult, the men reportedly gained new insights in life which helped to reshape their worldviews and life priorities [14]. In addition, previous experience with breast cancer in the family was associated with use of non-repressing coping styles (X^2^[1, *N* = 26] *r* = 5.60, *p* < 0.05). There was also a higher use of mature defence patterns (superior healthy neurotic functioning) in patients who use non-repressive coping [70]. Despite the identified active coping mechanisms, one study reported that majority (70%) of men with breast cancer used immature and neurotic defensive functioning and 53.8% used a repressive approach to bottle up their emotions and concerns and [70]:

*“I was kind of self-conscious the first year or so but um, I’m in pretty good shape, I’m relatively muscular, not super muscular, but I’m toned, I’m in shape, and I think a lot of times unless I’m really up close to people, I think a lot of times they don’t even see it… I’m not self-conscious. I go on vacation or go swimming at the beach, I don’t feel like people are staring at me.” *[67]* (p.38)*

*“Breast cancer, for me, means a whole complex of experiences, of realisations. It’s like being in the military, you know. You meet somebody who’s been in the military, you don’t have to say anything. But if you meet someone who hasn’t, there’s not a way in the world to describe what it’s like.” *[67]* (p.38)*

### Family support

Studies found that majority of patients (61.3–80%) disclosed and discussed their diagnosis with their spouses and close families while 4–21% refused to disclose or discuss with anyone [7, 13, 61]. This might be because less stigmatization was reported from close families and friends compared to broader social settings [32]. Such disclosure might also be protective as availability of marital support was found to influence treatment choice and outcomes. Men who were not currently married received chemotherapy significantly less often [52] and had significantly higher (in some cases up to 21%) mortality than married ones [52, 53].

This was corroborated by included qualitative studies which reported on the family support that men affected with breast cancer received. Spousal support was identified as a significant resource to seeking healthcare in the first instances as some wives had to push their partners to seek medical care [31, 57]. Spousal and family support also helped men to navigate through the breast cancer diagnosis, coming to terms with the disease [49, 57]. Family support was also an essential resource during the treatment and aftercare phase as family members offered emotional and practical support [47]:

*“My wife was my support – she and I talked about everything. At the beginning we talked about it and agreed that I would have her as my support and she would have her family to support her through. It worked well and I also got support from her family . . . mine were useless’’. *[13]* (p. 338).*

### Support from healthcare providers and other support groups

Studies reported the dimensions, contents and timing of information needs demonstrated by the patients. Men with breast cancer acknowledged the support received from healthcare providers regarding diagnosis, information, treatment options, and aftercare support [49, 57] with the most common source of information being verbal (92%), leaflets or booklets (53–71%) and internet (20%) [61]. Yet, 36–65% of participants felt their needs were not always met and wanted more information on various contents (particularly sexuality related information) at different times in their treatment (early/acute effects, late effects and ongoing quality of life) and in a more male specific manner [42].

Men with Breast cancer faced challenges in accessing needed support from healthcare facilities. Included studies reported experience of embarrassment and stigmatization within healthcare facilities where male breast cancer patients were meant to get support. 51.6% of patients experienced "extreme" or "very" severe embarrassment while waiting in the clinic among other female patients [13]. The experience of stigmatization was found to be higher within the cancer care system than other social surroundings with significantly higher stigmatization incidences reported in rehabilitation settings (mean = 1.50) and during hospitalisations (mean = 1.20) [53].

A mixed finding was observed regarding usage of peer supports. For one-to-one peer support, Iredale et al. (2006) reported low utilisation of formal support services with only 19% of participants speaking to other men who had breast cancer and only 1 in 4 indicating they would have liked that opportunity after their diagnosis. However, Midding et al. [53] found that more men (63.2%) had a one-on-one peer support from a female Breast Cancer Patient compared to 24.2% from another male breast cancer patient. This is consistent with the qualitative data which showed some men appreciated the opportunity to talk to other men with breast cancer on one-to-one basis [34, 71], other men did not prefer this and were satisfied with the support offered by the healthcare providers and their families [13]:

*‘‘…none of the guys wanted to have self-help groups ... I don’t think they need the psychological support that perhaps women do, and women tend to congregate and talk about these things anyway. I think this is, of course ... research I know ... but actually quite therapeutic in a way’’. *[13]* (p.338).*

*“To be honest, I don’t know how I would be managing if I had never had (the support group). They gave me back the will to live and I will always be grateful for that.” *[43]* (p. 9).*

In terms of group peer support, studies reported that only 15.3% of the participants were part of a peer support group and majority (96.3%) of participants who were not currently part of a support group did not wish to be part of a support group whether male only or mixed sex [53, 61].

## Discussion

Breast cancer is generally perceived to be a disease common among women albeit incidence among men is slowly rising, creating a need for health systems to be responsive to their needs. To this end, this review sought to develop a comparative understanding of the experiences of men with breast cancer and the treatment options available to them across different demographic settings. The review findings highlight the embodiment of breast cancer as a ‘feminine’ disease which is incongruent with what it means to be a ‘man’ and hegemonic masculinity discourses. Throughout the trajectory of the disease (that is, from diagnosis to aftercare), the review findings underscore the gendered nature of the disease with a lack of health system preparedness to support men who develop a disease perceived to be ‘feminine’. Though the treatment pathways were similar to those observed in the management of female breast cancer patients, they do not necessarily meet the unique needs of MBC across the disease trajectory warranting urgent attention considering the increasing prevalence of the disease among men. Male-specific treatment pathways, ongoing education, and professional support are also required.

The breast is seen as a symbol of femininity, and as incongruent with being male, together with the significant public health emphasis on the prevention of breast cancer among females [78, 79] have further championed the perception that breast cancer is a feminine illness [56, 67]. Thus, it was not surprising that the finding regarding being out of sync with one’s body resonated across the included studies. The breast cancer diagnosis which commenced the illness trajectory was really challenging for the men and filled with varied emotions. Despite the difficulty, the professional support available was often gendered and unsuitable to their needs. Thus, they mostly had to rely on their spouses and close families/ friends if they were able to open up to them, which may take some time. Coupled with the hegemonic masculinity ideology that a man must always be in charge and not demonstrate any emotions which can be perceived as weakness, it is likely that men will navigate through these on their own which can make the journey very lonely for them. Agreeing with a previous study, depressive symptoms, anxiety, and traumatic stress symptoms were common occurrences following the breast cancer diagnosis [43]. The culture of silence around the issue can lead to utilising avoidant coping mechanisms which may delay support seeking among men. Taken together, the findings highlight a need for tailor-made, individualised counselling support service for men before, during, and after breast cancer diagnosis. The need for healthcare professionals to consider the impact of the MBC on men cannot, therefore, be overemphasised.

Commencing treatment and aftercare/ rehabilitative support is an equally challenging phase for men living with breast cancer. A previous study has observed that gender impacts on the experience with breast cancer treatment [15]. The review findings highlighted the ‘feminised’’ nature of the treatment pathways with some practitioners not even knowing how to support the affected men. Information leaflets and other educational materials were generally noted to be filled with images of females which made the men feel out of place. Overall, these can serve as structural barriers which potentially deter men from seeking help even when required [34]. Undoubtedly, breast cancer affects more females than males. However, healthcare service delivery should be tailored to the unique needs of men to overcome the feeling of marginalisation or being left out. The impact of the therapeutic regimen should also be highlighted particularly as they can lead to loss of libido or erectile dysfunction which further diminishes one’s sense of being a man in relation to societal norms. Surgical procedures can lead to scars which serve as permanent reminders of the illness which can have life-long impact on men. Professional support should therefore not end after the diagnosis phase but should extend to the entire treatment continuum and aftercare. There is also a need to raise awareness of male breast cancer among healthcare practitioners to improve their approach to individuals through person-centred and male-specific care strategies. It may be worth reiterating the recommendation by Nguyen et al., [34] suggesting a guideline targeting men with breast cancer to support healthcare practitioners in the health and social service delivery process.

The need for support was reiterated throughout the review, and this is corroborated in a previous study where family and spousal support was critically important for men with advanced prostate cancer [80]. Interestingly, mixed findings were observed regarding the need for male-specific support groups. Although this may be based on individual preferences, it may also emanate from the hegemonic masculinity ideology [80, 81] or coping styles such as disengagement [20] as men may appear ‘stoic’ in the presence of such difficult moments and may not want to seek help [34, 82]. A breast cancer diagnosis can profoundly impact masculinity, with men grappling with navigating a threat to masculinity which collectively challenges one's sense of self and traditional gender roles [82–84].

Recent research shows changing perceptions of breast cancer as a "feminine disease" due to awareness campaigns and shifts in societal attitudes [85, 86]. Additionally, demographic factors like location of treatment, socioeconomic status, and age have been found to affect the quality of care and outcomes, while acknowledging the male breast cancer experience and its shared emotional aspects with women's experiences [87, 88]. These highlights evolving healthcare practices and societal norms regarding breast cancer.

Despite this, it is still cogent to understand their lived experiences and advocate for men support groups, if they would like to join one, as they navigate through the diagnosis, treatment, and aftercare pathway. This study presents the synthesis of multicultural evidence to highlight the cross-cultural similarity in the reaction and lived experience of men when faced with the diagnosis of breast cancer.

### Strengths and limitations

The strength of this mixed method is the inclusion of studies from different countries and settings in addition to including and synthesising studies on the experiences of patients with male breast cancer from diagnosis to aftercare. Notwithstanding, there are some limitations that need to be highlighted. Firstly, a real limitation of our review was including only studies published in English. Excluding studies that used a language other than English, potentially led to information loss that could come from relevant studies written in other languages and restricts this mixed methods review only to the views and perception of men living in English speaking countries or countries where practitioners write and publish in English. Secondly, we acknowledge that younger and older men may have unique experiences while navigating breast cancer diagnosis and treatment. These nuances were not captured in the current review and may be worth exploring in future studies.

## Conclusion

Men experience a myriad of issues following a breast cancer diagnosis, underscored by their ideology of masculinity. Our findings suggest the need for healthcare professionals’ training and education on managing interactions with MBC patients in a way that does not propagate a sense of awkwardness and otherness in a feminised support structure. Additionally, policy must address the structural barriers to treatment access for MBC including healthcare finance reimbursements that limit access to gendered specialist breast cancer treatments. Awareness creation efforts of MBC among the public as well as healthcare practitioners are urgently required to explain to the public through television programmes and awareness meetings that breast cancer is a disease like any other that affects both men and women. Creating such awareness could lead to changing the perception of men and promote early diagnosis, adherence to treatments, post-treatment monitoring, oncological results, and a better quality of life. Professional care intervention and support for MBC should not end after the diagnosis phase but should extend to the entire treatment continuum and aftercare. Preserving sexual function is an important finding highlighted from this review. Research will be needed to develop and test testosterone-preserving treatment modalities or optimising existing therapies in a way that is relevant to the priorities of MBC. This will also require the development of specialised guidelines for healthcare practitioners on MBC to optimise care and treatment for MBCs in a person-centred manner as suggested by other studies. To develop such individualised support frameworks, it is imperative to understand the specific needs, priorities, and support preferences among MBC patients.

## Data Availability

All data generated or analysed during this study are included in this published article.
